# Domain Organization of the UBX Domain Containing Protein 9 and Analysis of Its Interactions With the Homohexameric AAA + ATPase p97 (Valosin-Containing Protein)

**DOI:** 10.3389/fcell.2021.748860

**Published:** 2021-09-23

**Authors:** Jana Riehl, Ramesh Rijal, Leonie Nitz, Christoph S. Clemen, Andreas Hofmann, Ludwig Eichinger

**Affiliations:** ^1^Medical Faculty, Center for Biochemistry, Institute of Biochemistry I, University of Cologne, Cologne, Germany; ^2^Department of Biology, College Station, Texas A&M University, Texas, TX, United States; ^3^German Aerospace Center, Institute of Aerospace Medicine, Cologne, Germany; ^4^Medical Faculty, Center for Physiology and Pathophysiology, Institute of Vegetative Physiology, University of Cologne, Cologne, Germany; ^5^Department of Veterinary Biosciences, Melbourne Veterinary School, The University of Melbourne, Parkville, VIC, Australia

**Keywords:** p97/VCP/CDC48/TER/VAT ATPase, UBX domain containing protein 9 (UBXD9, TUG, ASPL, PUX1), IBMPFD (Inclusion Body Myopathy associated with Paget disease of bone and Fronto temporal Dementia), ALS (Amyotrophic Lateral Sclerosis), *Dictyostelium discoideum*, hexamer disassembly

## Abstract

The abundant homohexameric AAA + ATPase p97 (also known as valosin-containing protein, VCP) is highly conserved from *Dictyostelium discoideum* to human and a pivotal factor of cellular protein homeostasis as it catalyzes the unfolding of proteins. Owing to its fundamental function in protein quality control pathways, it is regulated by more than 30 cofactors, including the UBXD protein family, whose members all carry an Ubiquitin Regulatory X (UBX) domain that enables binding to p97. One member of this latter protein family is the largely uncharacterized UBX domain containing protein 9 (UBXD9). Here, we analyzed protein-protein interactions of *D. discoideum* UBXD9 with p97 using a series of N- and C-terminal truncation constructs and probed the UBXD9 interactome in *D. discoideum*. Pull-down assays revealed that the UBX domain (amino acids 384–466) is necessary and sufficient for p97 interactions and that the N-terminal extension of the UBX domain, which folds into a β_0_-α_–__1_-α_0_ lariat structure, is required for the dissociation of p97 hexamers. Functionally, this finding is reflected by strongly reduced ATPase activity of p97 upon addition of full length UBXD9 or UBXD9^261–573^. Results from Blue Native PAGE as well as structural model prediction suggest that hexamers of UBXD9 or UBXD9^261–573^ interact with p97 hexamers and disrupt the p97 subunit interactions via insertion of a helical lariat structure, presumably by destabilizing the p97 D1:D1’ intermolecular interface. We thus propose that UBXD9 regulates p97 activity *in vivo* by shifting the quaternary structure equilibrium from hexamers to monomers. Using three independent approaches, we further identified novel interaction partners of UBXD9, including glutamine synthetase type III as well as several actin-binding proteins. These findings suggest a role of UBXD9 in the organization of the actin cytoskeleton, and are in line with the hypothesized oligomerization-dependent mechanism of p97 regulation.

## Introduction

**FIGURE 1:**
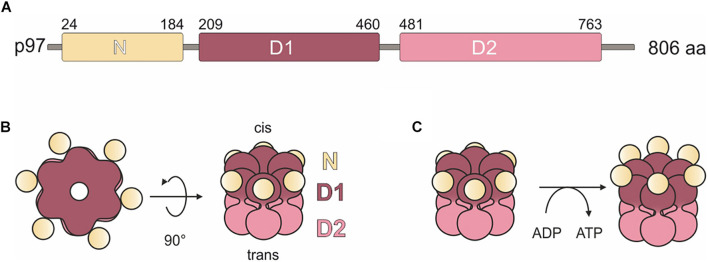
Domain organization and model of *H. sapiens* p97. **(A)** p97 domain organization. N, N domain (yellow); D1, ATPase domain D1 (purple); D2, ATPase domain D2 (pink). Numbers indicate amino acid positions. **(B)** Schematic representation of the structure of the p97 homohexamer. Each monomer comprises a globular N domain (N) depicted in yellow and two ATPase domains, D1 (purple) and D2 (pink), forming two stacked rings. Surrounded by the rings, a central pore forms, which extends from the *cis* side (D1 domain) through the entire protein to the *trans* side (D2 domain). **(C)** Conformational states of the N domains are dependent on the nucleotide bound state of the D1 domains. Left: bound ADP induces down-conformation. Right: bound ATP induces up-conformation.

The AAA + (ATPase Associated with diverse cellular Activities) ATPase p97, also known as valosin-containing protein (VCP), is a very abundant protein and evolutionarily highly conserved. It was first described in 1982 and has since emerged as a fundamental player in a plethora of cellular processes and signaling pathways (Moir et al., 1982; Madsen et al., 2009; van den Boom and Meyer, 2018). The protein has a tripartite structure comprising a globular N domain followed by the D1 and D2 domains that bind and hydrolyze ATP (Figure 1A; Wendler et al., 2012). p97 assembles into a ring shaped hexameric complex of six identical subunits, where the D1 and D2 domains form stacked rings with a “cis” and a “trans” side. The “cis” D1 domain is surrounded by the globular N domain (Figure 1B). Depending on nucleotide binding in the D1 domain, the N domain adopts an up- or down conformation (Figure 1C; Huyton et al., 2003; Bodnar and Rapoport, 2017; Banchenko et al., 2019).

For a long time, p97 was considered only as a segregase that extracts target proteins from complexes or membranes by ATP hydrolysis, so that they can be degraded by the proteasome (Ye, 2006). However, now it is clear that the fundamental function of p97 is the unfolding of proteins in numerous protein quality control pathways such as chromatin-associated degradation, the ubiquitin-proteasome system (UPS), mitochondria-associated degradation, endo-lysosomal damage response, macroautophagy, and endoplasmic-reticulum associated degradation (ERAD) (Bodnar and Rapoport, 2017; van den Boom and Meyer, 2018; Papadopoulos et al., 2020). The critical importance of p97 for cellular homeostasis is demonstrated by the lethality of knock-out mutations in unicellular and multicellular organisms (Müller et al., 2007). Furthermore, an increasing number of p97 missense mutations have been associated with various human diseases. Prominent examples include the late-onset and slowly progressive multi-system disorder Inclusion Body Myopathy associated with Paget disease of bone and Fronto temporal Dementia (IBMPFD) and Amyotrophic Lateral Sclerosis (ALS) (Watts et al., 2004; Johnson et al., 2010; Clemen et al., 2012; Evangelista et al., 2016). Since cancer cells have a heightened dependence on mechanisms of protein homeostasis, p97 is also a promising target for anti-cancer therapy (Deshaies, 2014).

To exert its locally, functionally and mechanically different tasks, p97 interacts with more than 30 different cofactors, most of which associate with the N domain. The p97 cofactors can be divided into three major classes, (i) substrate-recruiting cofactors, as e.g., the well-studied Ufd1/Npl4 heterodimer, (ii) substrate-processing cofactors as e.g., ubiquitin E3 ligases, and (iii) regulatory cofactors (Hänzelmann and Schindelin, 2017). The largest family of regulatory cofactors is the Ubiquitin Regulatory X (UBX) domain containing protein family, which has thirteen members in human and eleven in *D. discoideum* (Alexandru et al., 2008; Rijal et al., 2016). All members possess the eponymous, highly conserved UBX domain in their C-terminal region, which adopts a ubiquitin-like fold and is critical for the interaction with the N domain of p97 (Rezvani, 2016). Five of the *D. discoideum* UBXD family members, among them UBX domain containing protein 9 (UBXD9), are orthologs of human UBXD proteins (Rijal et al., 2016). Human UBXD9, also known as ASPL (alveolar soft part sarcoma locus) or TUG (tether containing a UBX domain for GLUT4), was originally identified in a genetic screen as part of an oncogenic fusion protein with the transcription factor TFE3 in alveolar soft part sarcoma cells (Ladanyi et al., 2001). The latter name, TUG, reflects its function in the redistribution of the glucose transporter GLUT4 in adipocytes (Bogan et al., 2003). In the basal state, UBXD9 traps GLUT4 storage vesicles (GSVs) at the Golgi apparatus. In response to insulin, UBXD9 is endoproteolytically cleaved by Usp25b, resulting in the release of the GSVs and their transport to the plasma membrane by the kinesin motor protein KIF5B (Habtemichael et al., 2018). Furthermore, it was shown that UBXD9 is required for the efficient re-assembly of the Golgi complex after brefeldin A removal (Orme and Bogan, 2012). A further UBXD9 homolog, PUX1 (plant UBX domain-containing protein 1), has been described in *Arabidopsis thaliana*. PUX1 is only about half the size and lacks the N-terminal half of UBXD9 (Rancour et al., 2004). Among all p97 cofactors described so far, *D. discoideum* UBXD9, human TUG and *A. thaliana* PUX1 are unique as they possess p97 hexamer disassembly activity (Rancour et al., 2004; Orme and Bogan, 2012; Rijal et al., 2016). In the following parts, we will refer to *D. discoideum* UBXD9 mostly just as UBXD9 and specify the orthologouss UBXD9 proteins by adding species names.

Here, we further analyzed UBXD9 by the characterization of the full-length protein and a series of N- and C-terminal truncation constructs with respect to their interactions with p97. Furthermore, to shed light on the UBXD9 interactome and further possible functions of UBXD9, we applied three different approaches to identify new interacting proteins.

## Materials and Methods

### Molecular Modeling

A search for *D. discoideum* UBXD9 (Dd-UBXD9) orthologs with known 3D structure using pGenThreader identified human ASPL (alveolar soft part sarcoma locus), also known as TUG (tether containing a UBX domain for GLUT4), UBXD9 and RCC17 (Hs-ASPL; PDB: 5ifs) as the best candidate (score: 86.243; next best score: 54.575). Therefore, a 3D model of Dd-UBXD9 was generated by comparative modeling using the software MODELLER (Sali and Blundell, 1993) and the structure-based amino acid sequence alignment of Dd-UBXD9 and Hs-ASPL (Supplementary Figure 1). Twenty different models were computed and the best one selected (lowest value of objective function: 980.54; next lowest value: 1044.5). Next, the modeled structures of *D. discoideum* p97 (VCP) (Rijal et al., 2016) and of *D. discoideum* UBXD9 (residues 320–522, 331–522, and 384–522) were used to generate a complex by aligning the partners to the structure of the human VCP (p97):ASPL complex (PDB: 5ifs) and adding ADP and Mg^2+^. The helical lariat of Dd-UBXD9 (residues 330–390) was manually adjusted by rigid body movements to remove any clashes with *D. discoideum* p97. The modeled complex was solvated and subjected to a molecular dynamics simulation for ∼5 ns simulation time using Gromacs (Abraham et al., 2015). Analysis of environmental (energy, temperature, pressure) as well as spatial parameters (e.g., distance between centers of gravity) showed that the model was stable throughout the simulation. Structure-based amino acid sequence alignments were generated with SBAL (Wang et al., 2012) based on predictions of secondary structure elements with PSIPRED (Bryson et al., 2005).

### Vector Construction

To generate GST-tagged UBXD9 polypeptides, the corresponding UBXD9 sequences were amplified by standard PCR technique from either AX2 cDNA or full-length UBXD9 cloned into the pBsr-C1-GFP vector (Rijal et al., 2016) using UBXD9 specific primers. The PCR products were cloned into the pGEX-6P-1 expression vector (GE Healthcare) and the sequences verified by sequencing. To generate the BirA-UBXD9 expression vector, the full-length UBXD9 coding sequence was amplified from AX2 cDNA and cloned into the previously described *D. discoideum* BirA expression vector (Batsios et al., 2016) via the *Sac*I and *Bam*HI restriction sites. The construct contained a linker encoding YKGGSGGSGGSRLREL between BirA and UBXD9. The insert of the resulting expression plasmid was verified by sequencing.

### *D. discoideum* Culture and Transformation

*D. discoideum* strains were grown in AX2 medium (for 1 l: 14.3 g bacteriological peptone, 7.15 g yeast extract, 18 g maltose, 0.62 g Na_2_HPO_4_ 2H_2_O, 0.49 g KH_2_PO_4_, pH 6.7) at 21°C either on Petri dishes (Ø 100 mm) or in suspension in Erlenmeyer flasks with shaking at 160 rpm (Brink et al., 1990) or on a lawn of *Klebsiella aerogenes* on SM agar plates (Williams and Newell, 1976). Recombinant strains were cultured under selective pressure in the presence of 5 μg/ml blasticidin S. For cell biological and biochemical experiments, log phase cells at 2–4 × 10^6^ cells/ml were used. The BirA-UBXD9 expression plasmid was transformed into wild-type AX2 cells by electroporation (Gaudet et al., 2007) and transformants were selected with 5 μg/ml blasticidin S. Stable transformants expressing BirA-UBXD9 were verified by PCR and Western blot analysis. The strains expressing GFP-UBXD9 or UBXD9-GFP have been described previously (Rijal et al., 2016).

### Purification of Recombinant Proteins and Immunoblotting

Purification of GST-tagged full-length and truncated UBXD9 proteins and of p97 from either *E. coli* XL1 Blue cells (New England Biolabs) or ArcticExpress cells (Stratagene GmbH) was performed as described previously (Rijal et al., 2016). For biochemical assays, the purified GST-fusion proteins were either used bound to Glutathione-Agarose 4B beads (MACHEREY-NAGEL GmbH) or the recombinant protein was liberated from the beads by cleavage with 5 U/ml PreScission protease (GE Healthcare GmbH) for 16 h at 4°C on a rotating wheel. Protein concentrations were determined by the Bradford assay (Bradford, 1976). For quality control of purified proteins and GST-fusion protein bound beads, SDS-PAGE (Laemmli, 1970) followed by Coomassie Brilliant Blue staining or Western blotting (Towbin et al., 1979) was performed. Full-length and truncated UBXD9 proteins were detected with rabbit polyclonal UBX23520 antibody at a 1:20,000 dilution (Rijal et al., 2016), p97 was detected with rabbit polyclonal p97_8_6841 antibody at 1:10,000 dilution (Arhzaouy et al., 2012), GFP with mouse monoclonal K3-184-2 antibody at a 1:50 dilution (Noegel et al., 2004), actin with the monoclonal Act1-7 antibody at a 1:40 dilution (Simpson et al., 1984), ubiquitin with mouse monoclonal P4D1 antibody at a 1:1,000 dilution (Cell Signaling Technology), and BirA with a rabbit polyclonal antibody at a 1:5,000 dilution (kindly provided by Dr. Ralph Gräf, University of Potsdam). Secondary antibodies used were anti-rabbit and anti-mouse IgG conjugated with horseradish peroxidase (HRP) at a 1:10,000 dilution (Sigma-Aldrich Corp.). Detection was done by chemiluminescence using the SuperSignal West Pico PLUS chemiluminescent substrate (Thermo Fisher Scientific Inc.) in conjunction with the Intas ECL Chemostar documentation system. The software LabImage 1D L-340 (Intas Science Imaging Instruments GmbH) was used for the determination of molecular masses and the quantification of the detected proteins.

### Co-immunoprecipitation

Co-immunoprecipitation experiments for the identification of novel UBXD9 interaction partners were performed with log phase AX2 cells (2–4 × 10^6^ cells/ml) ectopically expressing UBXD9-GFP, GFP-UBXD9, or GFP (negative control). 1 × 10^8^ cells were harvested (500 × g, 5 min), washed twice with Soerensen phosphate buffer (14.6 mM KH_2_PO_4_, 2.0 mM KH_2_PO_4_, pH 6.0) and the cell pellet shock frozen with liquid nitrogen. Pellets were resuspended in 1 ml lysis buffer (20 mM Tris/HCl pH 7.5, 100 mM NaCl, 1 mM DTT, 20 mM MgCl_2_, 5% glycerol, 1 mM benzamidine, 10 μg/ml aprotinin/leupeptin, 1:50 proteinase inhibitor cocktail (Roche), 1:100 PEFA block) followed by centrifugation at 20,000 × *g* for 10 min. The supernatant, containing soluble proteins, was incubated with GFP-trap beads (Chromotek) for 2 h at 4°C. Beads were washed four times with wash buffer I (50 mM Tris/HCl pH 7.5, 150 mM NaCl, 1 mM DTT, 0.2% NP-40) and twice with wash buffer II (50 mM Tris/HCl pH 7.5, 150 mM NaCl, 1 mM DTT). Bound proteins were either analyzed by SDS-PAGE and Coomassie Brilliant Blue staining or further processed for mass spectrometry.

### Proximity-Dependent Biotin Identification

The BioID method was essentially performed as described (Batsios et al., 2016). Briefly, log phase BirA-UBXD9 expressing AX2 cells were cultured in the presence of 50 μM Biotin for 16 h. 2 × 10^8^ cells were harvested and the cells further processed as for co-immunoprecipitation. Soluble proteins were incubated with streptavidin-coupled sepharose beads (GE Healthcare GmbH) for 2 h at 4°C. After washing of the beads (see procedure for co-immunoprecipitation) bound proteins were either analyzed by Western blot analysis or further processed for mass spectrometry.

### Mass Spectrometry

Mass spectrometry was carried out at the CECAD/CMMC Proteomics Facility (University of Cologne). Samples were prepared by the in-solution digestion of proteins and StageTip purification of peptides according to the protocol of the facility^1^. The samples were analyzed using an EASY nLC 1,000 UPLC (Thermo Fisher Scientific) coupled to a Q-Exactive Plus (Thermo Scientific) mass spectrometer. Peptides were loaded with solvent A (0.1% formic acid in water) onto an in-house packed analytical column (50 cm × 75 μm I.D., packed with 2.7 μm C18 Poroshell beads, Agilent) and were chromatographically separated. The mass spectrometer was operated in data-dependent acquisition mode, where the Orbitrap acquired full MS scans (300–1750 *m/z*) at a resolution of 70,000 with an automated gain control (AGC) target of 3 × 10^6^ ions collected with 20 ms. Precursors were dynamically excluded for 20 s. The ten most intense peaks were subjected to HCD fragmentation. All mass spectrometric raw data were processed with Maxquant (version 1.5.3.8) and its implemented Andromeda search engine (Cox et al., 2011). Two-sample two-tailed Student’s *t*-test were performed in Perseus (version 1.6.5) for pairwise comparisons. Proteins with a fold change > 1.4 and a *p* < 0.05 in the experiment versus negative control were considered to represent putative UBXD9 interacting proteins. The mass spectrometry proteomics data have been deposited to the ProteomeXchange Consortium^2^ via the PRIDE (Perez-Riverol et al., 2019) partner repository with the dataset identifiers PXD027160 and PXD027162. Four independent biological replicates were performed.

### Miscellaneous Methods

Blue Native-PAGE (BN-PAGE) was performed for the analysis of native protein complexes. Purified recombinant proteins in 1 × PBS, 1 mM DTT, 1 mM ATP were mixed with 4 × NativePAGE Sample Buffer (Thermo Fisher Scientific Inc.) followed by separation in Invitrogen Novex Native-PAGE 4–16% Bis-Tris Protein-Gels (Thermo Fisher Scientific Inc.). Electrophoresis was performed in the cold-room with pre-chilled buffers (cathode buffer: 50 mM Tricine, 15 mM Bis-Tris pH 7.0, 0.02% Coomassie G-250; anode buffer: 50 mM Bis-Tris pH 7.0) at 150 V for 4 h. Gels were stained with Coomassie Brilliant Blue and protein molecular masses were determined based on the NativeMark protein standard (Invitrogen). The ATPase activity assay for p97 either alone or in the presence of full-length or truncated UBXD9 proteins was carried out in phosphate-free sample buffer (10 mM Tris/HCl pH 7.4, 100 mM NaCl, 1 mM DTT, 1 mM MgCl_2_) in the presence of 1 mM ATP according to the instructions of the manufacturer (Enzo Life Sciences, Farmington, NY). The ATPase activity of p97 was calculated based on the amount of the generated free phosphate. Sucrose density gradient sedimentation and pull-down assays were performed as described (Rijal et al., 2016).

## Results

### Ubiquitin Regulatory X Domain Containing Protein 9 Is Highly Conserved

**FIGURE 2:**
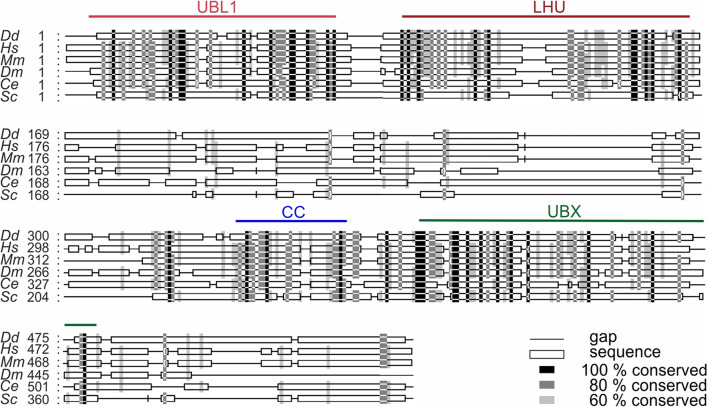
Sequence alignment of UBXD9 proteins from different model organisms. A multiple sequence alignment of UBXD9 protein sequences from *D. discoideum* (*Dd), H. sapiens (Hs), Mus musculus (Mm), Drosophila melanogaster (Dm), Caenorhabditis elegans (Ce), and S. cerevisiae (Sc)* was generated with Clustal Omega (Version 1.2.4; Goujon et al., 2010; Sievers et al., 2011) and then edited with Gendoc (v0.7.2). Genbank accession numbers are: (*Dd*) XP_641771, (Hs) NP_076988, (*Mm*) NP_081153, (*Dm*) NP_001027152, (*Ce*) NP_505652, (*Sc*) EWG89362. Shading reflects sequence conservation. The domain organization is homologous in all species and sequence conservation is high in the specified domains. UBL1, Ubiquitin-like domain 1; LHU, Low Homology UBX domain; CC, Coiled coil domain; UBX, Ubiquitin regulatory X domain. Numbers indicate amino acid positions.

**FIGURE 3:**
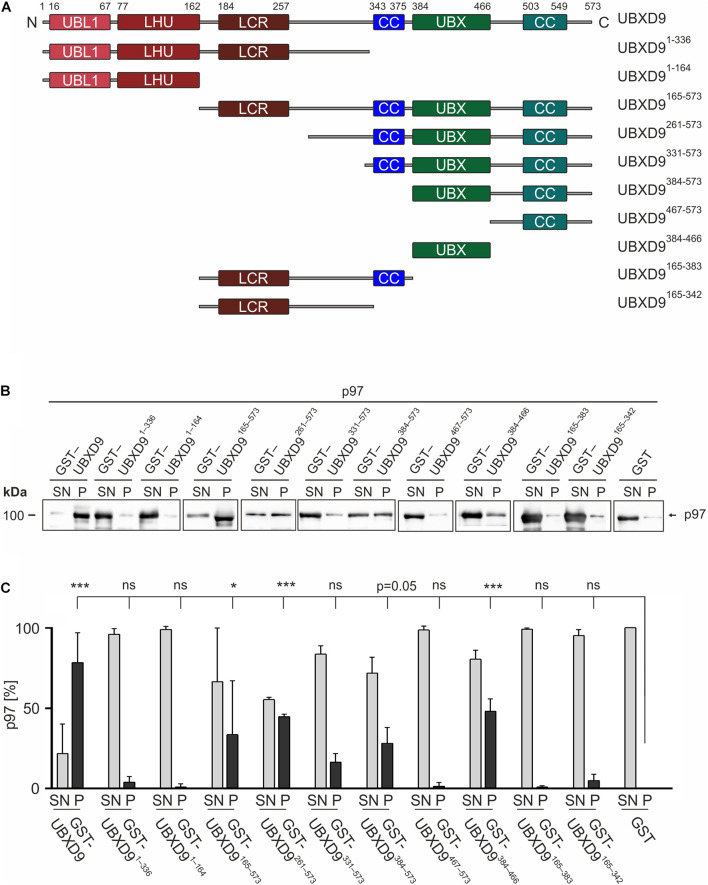
The UBX domain is necessary and sufficient to bind to p97. **(A)** Schematic representation of the *D. discoideum* UBXD9 domain organization and of the analyzed UBXD9 truncation constructs. N, N-terminus; C, C-terminus; LCR, Low complexity region, UBL1, Ubiquitin-like domain 1; LHU, Low Homology UBX domain; CC, Coiled coil domain; UBX, Ubiquitin regulatory X domain. Numbers indicate amino acid positions of UBXD9. **(B)** Pull-down experiments with recombinant p97 and full-length or truncated GST-UBXD9 coupled to glutathione beads. GST coupled to glutathione beads was used as negative control. Representative Western blots with anti p97 antibodies of supernatants (SN) and pellets (P) are shown. The position of p97 is indicated. **(C)** Quantification of p97 in the SN and P fractions. The sum of the values of SN and P for each experiment was taken as 100%. The bar graphs represent mean values and standard deviations (SD) of at least three independent experiments. For statistical analysis, the Dunnett’s multiple comparison test, implemented in GraphPad Prism as *post hoc* analysis, was performed. ***p ≤ 0.001; *p ≤ 0.05. ns, not significant.

UBXD9 is a member of the ubiquitin X domain (UBXD) protein family, which makes up the largest subgroup of p97 cofactors. The UBXD9 domain structure is highly conserved across different eukaryotes and is composed of the N-terminal ubiquitin-like (UBL1) and the low homology UBX (LHU) domains followed by a coiled coil domain in the middle part. The C-terminal region harbors the eponymous UBX domain, which is present in and characteristic for all members of the UBXD protein family (Figure 2). In addition, we identified in *D. discoideum* UBXD9 a low complexity region (LCR) located between the LHU and coiled coil domains, and a second coiled coil domain in the C-terminal region (Figure 3A). The SHP box, a further p97 binding motif, which was identified in human and mouse UBXD9 (Yeung et al., 2008; Orme and Bogan, 2012), is not present in *Drosophila melanogaster*, *Caenorhabditis elegans*, *Saccharomyces cerevisiae*, and *D. discoideum* UBXD9 (not shown). We previously reported the direct interaction of UBXD9 with p97 (Rijal et al., 2016). To analyze the contribution of the different domains of UBXD9 to the interaction with p97 and to narrow down interacting regions, we generated a series of C- and/or N-terminal UBXD9 truncation constructs. The domain structures of full-length *D. discoideum* UBXD9 and of the generated truncation constructs are displayed in Figure 3A.

### The Ubiquitin Regulatory X Domain Is Necessary and Sufficient for the Interaction With p97

To elucidate the p97 binding region(s) of UBXD9 we performed pull-down experiments with recombinant p97 and full-length UBXD9 and the different UBXD9 truncation constructs tagged with GST (Figure 3A). In the presence of full-length UBXD9 most of p97 was found in the pellet fraction of glutathione sepharose beads (Figure 3B, left two lanes), while most of p97 remained in the supernatant in the presence of GST (Figure 3B, right two lanes). This result confirmed the specific binding of full-length UBXD9 to p97 (Rijal et al., 2016). Deletion of the C-terminal part of UBXD9 in the N-terminal constructs, UBXD9^1–336^ and UBXD9^1–164^, resulted in a loss of p97 binding activity (Figure 3B, lanes 3–6 from left). Similarly, the truncation constructs UBXD9^467–573^, UBXD9^165–383^, and UBXD9^165–342^, which all lack the UBX domain, were also not able to bind p97 and almost all p97 remained in the supernatant (Figure 3B, lanes 15–16 and 19–22 from left). In contrast, the UBXD9 truncation constructs UBXD9^165–573^, UBXD9^261–573^, UBXD9^331–573^, UBXD9^384–573^, and UBXD9^384–466^, which all harbor the UBX domain, were all able to bind p97 (Figure 3B, lanes 7–14 and 17–18 from left). The shortest construct sufficient to bind p97 was UBXD9^384–466^, which comprises the UBX domain only. SDS-PAGE followed by Coomassie staining showed the presence of GST, GST-tagged full-length UBXD9, and all GST-UBXD9 truncation constructs in the pellet fraction (Supplementary Figure 2). Quantification of p97 in the supernatant and pellet fractions of three independent experiments revealed strongest binding by full length UBXD9, followed by UBXD9^261–573^, UBXD9^384–466^, UBXD9^165–573^, and UBXD9^384–573^ (Figure 3C). Quantification also revealed that the interaction of UBXD9^331–573^ with p97 was not significant, although the shorter UBXD9^384–573^ and UBXD9^384–466^ clearly pulled down p97 (Figure 3C). We assume that the amino acids 331–383 in this construct somehow interfere with binding of the UBX domain to p97. In summary, these results confirm the interaction of UBXD9 with p97 and show that the UBX domain is necessary and sufficient for binding to p97.

### The Presence of a UBX Domain Containing Protein 9 Lariat Structure Is Required for the Disassembly of p97 Hexamers

**FIGURE 4:**
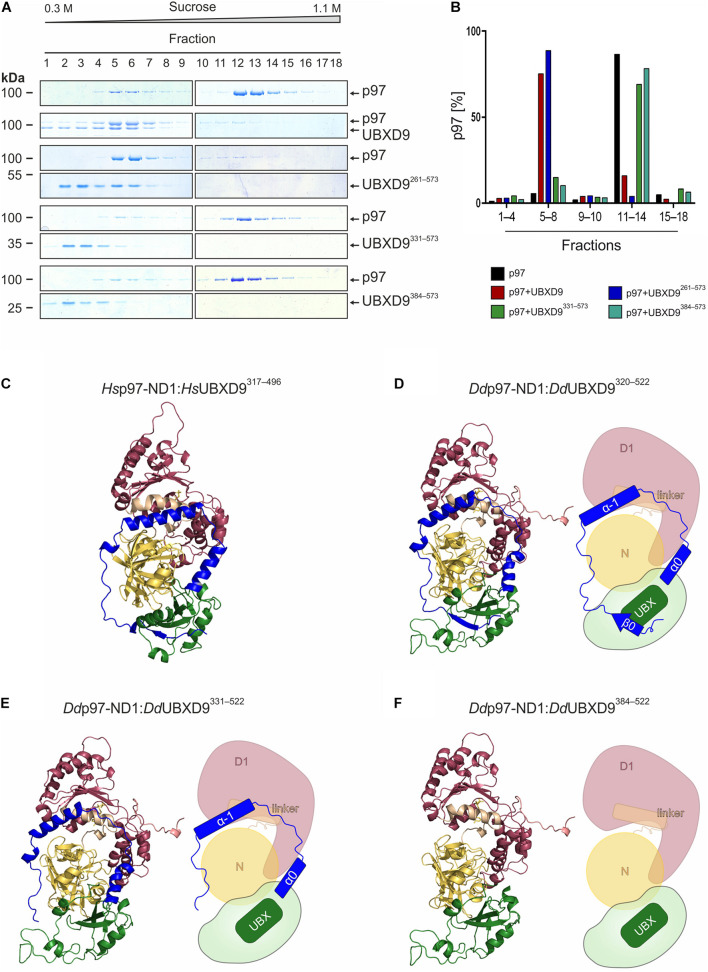
The helical lariat structure of UBXD9 is pivotal for its disassembly activity. **(A)** Sucrose density gradient sedimentation with p97 alone (top panel) or with equimolar amounts of p97 and full-length UBXD9 (2nd panel from top) or p97 and UBXD9^261–573^ (3rd and 4th panels from top), or p97 and UBXD9^331–573^ (5th and 6th panels from top), or p97 and UBXD9^384–573^ (lower two panels). Before loading the samples on the sucrose density gradient (0.3–1.1 M sucrose), they were incubated with 1 mM ATP for 30 min. Fractions were removed consecutively after centrifugation and analyzed by SDS-PAGE and Coomassie Brilliant Blue staining. p97 monomers or dimers are present in fractions 5–8 and p97 hexamers in fractions 11–14. Fractions are numbered, starting with #1 at 0.3 M sucrose. The positions of p97, full-length UBXD9 and UBXD9 truncation constructs are indicated. **(B)** Bar graphs depicting the quantification of p97 in fractions 1–4, 5–8, 9–10, 11–14, and 15–18 of the different experiments. The bar graphs represent mean values of at least three independent experiments. The determined total amount of p97 in all 18 fractions was set to 100%. **(C)** Structural model of the *Hs* p97-ND1:Hs UBXD9^317–496^ (VCP:ASPL; PDB: 5ifs) complex. UBXD9^317–496^ is shown in green and ochre and the ND1 domains of p97 (VCP) are colored yellow and dark pink, respectively. The region N-terminal of the UBX domain forms a loop, which encompasses two α-helices (α_–__1_ and α_0_; blue), an extended linker (blue) and a β-strand (β_0_, blue). The α-helices and the linker embrace the N domain of p97 and the β_0_-strand appears to be crucial for the closure of the lariat structure. The helical lariat probably interferes with the D1:D1’ inter-monomeric interaction of the p97 hexamer, causing its disassembly (Arumughan et al., 2016; Banchenko et al., 2019). **(D–F)** Homology models and schematic representation of the *D. discoideum* p97-ND1:UBXD9^320–522^ **(D)**, p97-ND1:UBXD9^331–522^ **(E)**, and p97-ND1:UBXD9^384–522^ **(F)** complexes representing our truncation constructs UBXD9^261–573^, UBXD9^331–573^, and UBXD9^384–573^, respectively. Since our modeling was based on the *Hs-*p97-ND1:*Hs-*UBXD9^317–496^ (VCP:ASPL; PDB: 5ifs) complex, the structural models could only be generated from amino acids 320–522 of *D. discoideum* UBXD9.

We used sucrose density gradient centrifugation in combination with SDS-PAGE to monitor the oligomeric status of p97 alone or in the presence of full-length UBXD9 and various UBXD9 truncation constructs. It has previously been shown that full length *D. discoideum* UBXD9, human UBXD9 (also termed TUG, ASPL, RCC17) and *A. thaliana* UBXD9 (termed PUX1, plant UBX domain-containing protein 1) have the ability to disassemble p97 hexamers (Rancour et al., 2004; Orme and Bogan, 2012; Rijal et al., 2016). Analysis of p97 alone showed the presence of most p97 in higher sucrose density fractions, representing p97 hexamers (Figure 4A top, fraction 12–14). Only a small amount of p97 was present in lower sucrose density fractions, representing monomers or dimers (Figure 4A top, fractions 5–7). A similar result as for p97 alone was obtained when p97 was incubated with either UBXD9^1–336^ (data not shown), UBXD9^331–573^, or UBXD9^384–573^ (Figure 4A, four panels from bottom). In contrast, full-length UBXD9 as well as UBXD9^261–573^ were able to disassemble p97 hexamers, as represented by the shift of p97 from high to low molar sucrose fractions (Figure 4A, second to fourth panel from top). Quantification of three independent experiments confirmed that the p97 distribution in the presence of UBXD9^331–573^ and UBXD9^384–573^ was comparable to p97 alone and that only full-length UBXD9 and UBXD9^261–573^ were able to disassemble p97 hexamers (Figure 4B). Of note, the ATPase activity of p97 is high in the hexameric state and low for p97 monomers (Rijal et al., 2016). In support of the p97 hexamer disassembly activity of UBXD9 and UBXD9^261–573^, we measured in their presence a strong reduction of the p97 ATPase activity (Supplementary Figure 3).

As the three UBXD9 truncation constructs UBXD9^261–573^, UBXD9^331–573^, and UBXD9^384–573^ had the ability to bind p97 (Figures 3B,C), we next were interested in their structural differences. Recently, the crystal structure of a *H. sapiens* UBXD9 truncation construct encompassing amino acids 317–497 in complex with the N and D1 domains of p97 was solved (Arumughan et al., 2016). The 3D structure of the UBXD9 truncation construct revealed a helical lariat, comprising the α-helices α_–__1_, and α_0_ and an unordered region, which embraces the N domain of p97. Upstream of the unordered region a short beta strand (β_0_) closes the helical lariat (Figure 4C). Only a construct with the extended UBX domain harboring the complete β_0_-α_–__1_-α_0_ lariat, as present in *H. sapiens* UBXD9^317–497^, had the ability to disassemble p97 hexamers (Arumughan et al., 2016). A structure-based amino acid sequence alignment of *D. discoideum* UBXD9 and *H. sapiens* UBXD9 revealed that the secondary structure elements of the helical lariat are conserved in *D. discoideum* UBXD9 (Supplementary Figure 1). We next performed comparative modeling with the *D. discoideum* N and D1 domains of p97 and the UBXD9 truncation constructs UBXD9^320–522^, UBXD9^331–522^, and UBXD9^384–522^, respectively (Figures 4D–F). Since our modeling was based on the published *H. sapiens* UBXD9^317–497^ structure, we could not model the regions encompassing amino acids 261–319 and 523–573 of the constructs UBXD9^261–573^, UBXD9^331–573^, and UBXD9^384–573^, which we had analyzed for p97 hexamer disassembling activity. The results showed that only UBXD9^261–573^ carries the complete lariat (Figure 4D), whereas UBXD9^331–573^ lacks the closing beta-strand β_0_ (Figure 4E) and UBXD9^384–573^ the complete lariat (Figure 4F). Thus, we conclude that the complete lariat is required for the dissociation of p97 hexamers. The N-terminal β_0_ strand appears to be crucial as it mediates the closure of the helical lariat. Its absence likely weakens the lariat structure and results in an inability to disassemble the p97 hexamer.

### UBX Domain Containing Protein 9 Forms Oligomers

**FIGURE 5:**
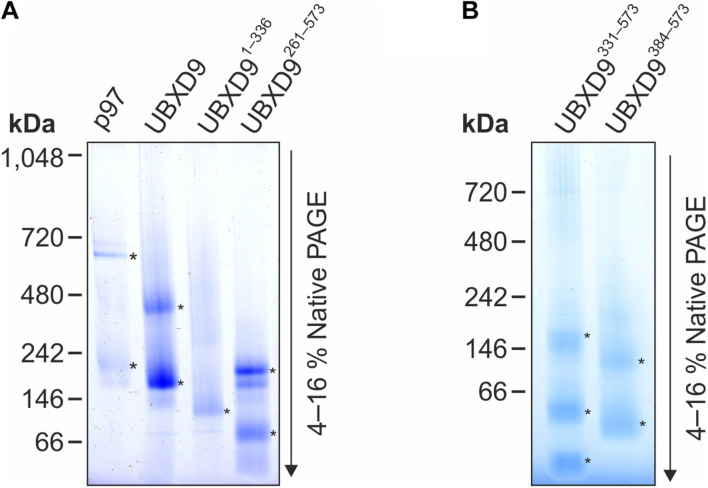
Blue Native PAGE analysis of p97, full-length UBXD9 as well as UBXD9 truncation constructs. Representative Blue Native PAGE gels of purified recombinant non-tagged *D. discoideum* proteins. Specific bands are marked by asterisks. **(A)** p97, UBXD9, UBXD9^1–336^ and UBXD9^261–573^. **(B)** UBXD9^331–573^ and UBXD9^384–573^.

Since *D. discoideum* UBXD9 contains two coiled coil regions (aa 343–375 and 503–549) and interacts with the hexameric p97, we analyzed by Blue Native PAGE whether UBXD9 forms multimers. We used p97 as positive control and, as expected, we observed a band of about 600 kDa, representing the p97 hexamer. In addition, a band of about 200 kDa was visible, corresponding in size to a p97 dimer (Figure 5A, lane 1). Analysis of UBXD9, UBXD9^261–573^, UBXD9^331–573^, and UBXD9^384–573^ (GST-tag cleaved for all) revealed for each protein two major bands. Based on the calculated molecular masses of approximately 65 kDa for UBXD9, 37 kDa for UBXD9^261–573^, 29 kDa for UBXD9^331–573^, and 23 kDa for UBXD9^384–573^, the upper band of each protein corresponds in size to a hexamer and the lower band to either a dimer or trimer (Figure 5A, lanes 2 and 4, Figure 5B). For UBXD9^331–573^ we detected a third band of approximately 30 kDa, which corresponds in size to a monomer (Figure 5B, lane 1). We also analyzed the N-terminal construct UBXD9^1–336^ and detected only a single band of around 120 kDa, corresponding in size to a trimer (Figure 5A, lane 3). We do not have an explanation for the possible trimer formation of this construct, as it does not contain the N-terminal coiled coil moiety (aa 343–375) and as the complete N-terminal region of UBXD9 was dispensable for hexamer formation of the other constructs. In summary, our results show, that full-length UBXD9 and the analyzed truncation constructs form dimers or trimers and hexamers. Since all constructs for which multimer formation was observed contained the C-terminal coiled coil domain (aa 503–549), we conclude that this domain is responsible for the formation of UBXD9 hexamers. The results of our biochemical analyses of full-length UBXD9 and UBXD9 truncation constructs are summarized in Table 1.

**TABLE 1:** Summary of biochemical properties of full length UBXD9 and UBXD9 truncation constructs.

**Construct**	**MM (kDa)**	**Hexamer formation**	**p97 binding**	**p97 Hexamer disassembly**
UBXD9	65	Yes	Yes	Yes
UBXD9^1–336^	37	No	No	No*
UBXD9^1–164^	19	nd	No	nd
UBXD9^165–573^	47	nd	Yes	nd
UBXD9^261–573^	37	Yes	Yes	Yes
UBXD9^331–573^	29	Yes	Yes	No
UBXD9^384–573^	23	Yes	Yes	No
UBXD9^467–573^	13	nd	No	nd
UBXD9^384–466^	10	nd	Yes	nd
UBXD9^165–383^	25	nd	No	nd
UBXD9^165–342^	20	nd	No	nd

MM, molecular mass (rounded); nd, not determined; *, not shown.

### UBX Domain Containing Protein 9 Interactome Mapping Confirms Interaction With p97 and Identifies Novel Interaction Partners

**FIGURE 6:**
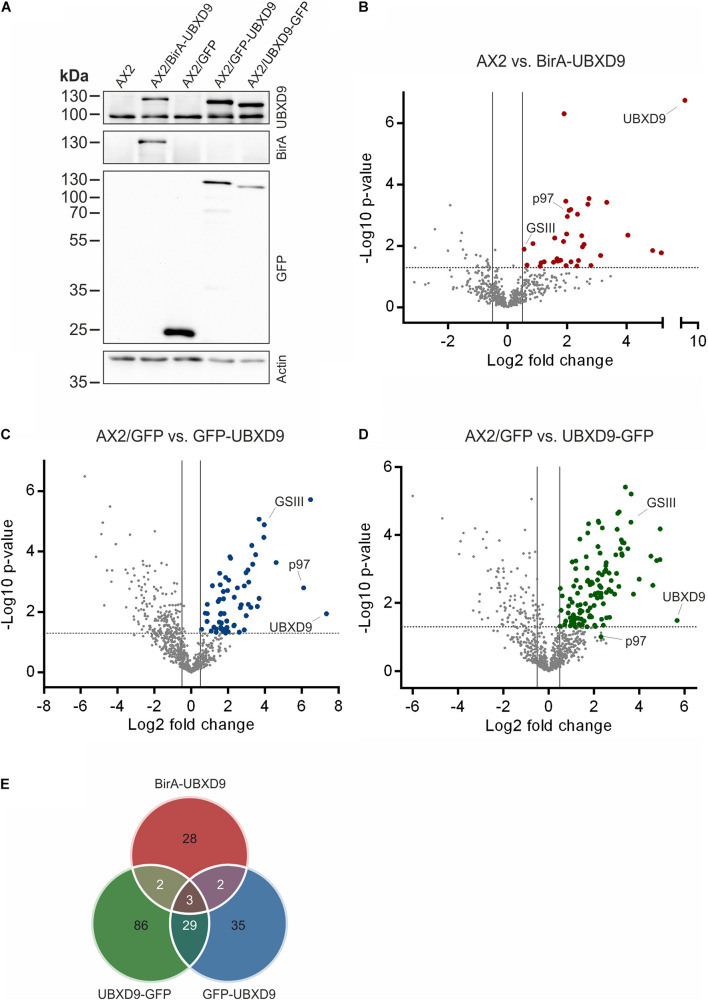
Interaction proteomics. **(A)** Verification of the BirA-UBXD9, GFP, GFP-UBXD9, and UBXD9-GFP expressing strains by western blotting using the polyclonal UBXD9 (top panel), the polyclonal BirA (2nd panel from top), and the monoclonal GFP (3rd panel from top) antibodies. Detection of actin (bottom panel) with the monoclonal actin antibody was used as loading control. **(B–D)** Volcano plots depicting *p*-value versus fold change (FC) for all proteins identified by mass spectrometry in AX2 versus AX2/BirA-UBXD9 expressing cells **(B)**, AX2/GFP versus AX2/GFP-UBXD9 expressing cells **(C)** and AX2/GFP versus AX2/UBXD9-GFP expressing cells **(D)**. Putative UBXD9 interacting proteins with a fold change ≥ 1.4 and a *p* ≤ 0.05 are depicted as red, blue or green dots, respectively. Dots representing UBXD9, p97, and glutamine synthetase type III (GSIII) are indicated. The plots were done using GraphPad prism. **(E)** Venn diagram of significantly enriched proteins in the pull-down experiments of AX2/BirA-UBXD9 (red), AX2/GFP-UBXD9 (blue), and AX2/UBXD9-GFP (green) cells. The intersections of the circles provide the number of proteins that were identified in two or all three approaches. Only those proteins with a fold change ≥ 1.4 and a *p* ≤ 0.05 were used as input.

**FIGURE 7:**
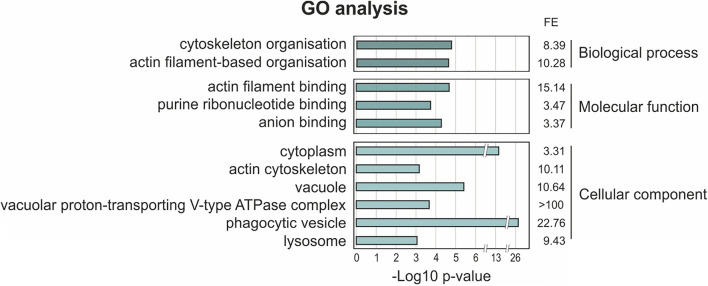
Gene ontology (GO) term enrichment analysis of proteins identified as putative interaction partners of UBXD9 in at least two experimental setups. The proteins, which were identified in at least two experimental approaches (listed in Table 2), were used as input for GO analysis. The analysis was performed with PANTHER version 15.0. For the enriched categories –Log10 *p*-value and fold enrichment (FE) are given.

In order to map the UBXD9 interactome, we employed two experimental approaches. We used AX2 strains that express GFP-UBXD9 or UBXD9-GFP (Rijal et al., 2016) and generated a new BirA-UBXD9 expressing strain for the identification of UBXD9 interacting proteins. The latter strain was used for proximity-dependent biotin identification (BioID), which has become a powerful tool to identify protein-protein interactions (Roux et al., 2012; Batsios et al., 2016). In this assay, the bait protein is fused to the R118G-mutated BirA biotinylase from *E. coli*. Interacting proteins will get biotinylated and can then be isolated through binding to streptavidin-coupled sepharose beads. We first confirmed expression of UBXD9 and the respective fusion proteins in our strains by Western blot analysis of total cell lysates with antibodies against UBXD9, BirA, and GFP. Equal loading was verified by staining with an actin antibody (Figure 6A). Next, we performed immune-precipitation (IP) experiments and analyzed the proteins bound to the GFP-trap or streptavidin-sepharose beads by silver staining and Western blotting (Supplementary Figure 4). Silver staining revealed weak bands corresponding in size to GFP-UBXD9 and BirA-UBXD9. The identity of these bands was confirmed by immunodetection with the UBXD9 antibody. It also revealed the presence of endogenous UBXD9 in the precipitates via binding to the fusion proteins, supporting the conclusion from Blue Native PAGE that UBXD9 can undergo homo-oligomerization (Figure 5 and Supplementary Figure 4). For mass-spectrometric identification of bound proteins we performed for each approach four independent experiments. To visualize differentially expressed proteins we generated volcano plots of the identified proteins in AX2 versus AX2/BirA-UBXD9, and AX2/GFP versus AX2/UBXD9-GFP and AX2/GFP-UBXD9 (Figures 6B–D). Proteins with a fold change ≥ 1.4 and a *p* ≤ 0.05 were defined as significantly enriched in the BirA-UBXD9, GFP-UBXD9, or UBXD9-GFP expressing strains and are depicted as red, blue or green dots, respectively. Using these criteria we identified 35 putative UBXD9 interacting proteins in the BioID approach, 69 with N-terminally tagged UBXD9 and 120 with C-terminally tagged UBXD9 (Supplementary Tables 1–3). We applied Venny to identify common enriched proteins in our three approaches and found 32 common proteins between GFP-UBXD9 and UBXD9-GFP, five between GFP-UBXD9 and BirA-UBXD9, and five between UBXD9-GFP and BirA-UBXD9 expressing strains. Only three proteins, namely p97, UBXD9 and the putative glutamine synthetase type III (GSIII) were found in all three approaches (Figure 6E and Table 2). The mode of interaction between UBXD9 and GSIII and its cellular function is currently unknown. Those proteins which were identified in at least two of the three approaches are of particular interest as they are strong candidates for further novel UBXD9 interacting proteins (Figure 6E and Table 2). Among these proteins, we found the ER resident Grp78 (luminal-binding protein 2, Bip), which could link UBXD9 to the ER stress response. To elucidate possible enrichments of biological processes, molecular functions, and cellular components among these proteins, we performed a gene ontology (GO) analysis with PANTHER (Mi et al., 2021). In the biological process category, we found “cytoskeleton” and “actin filament-based organization” significantly enriched (Figure 7). This was reflected in the molecular function and cellular component categories by an enrichment of “actin filament binding” and “actin cytoskeleton” as well as “phagocytic vesicle” (Figure 7). Thus, GO analysis points to a connection of UBXD9 with the actin cytoskeleton. Indeed, of the 36 proteins that were found enriched in at least two of our approaches seven are classified as cytoskeletal proteins (Table 2, bold entries). The nature of this observation will have to be investigated in future studies.

**TABLE 2:** List of putative interacting proteins, which were identified in at least two different approaches.

**Protein name**	**DDB_G ID**	**BirA-UBXD9**		**UBXD9-GFP**		**GFP-UBXD9**	
		**FC**	**P**	**FC**	**P**	**FC**	**P**
UBXD9	DDB_G0279285	9.23	6.75	5.67	1.49	7.34	1.94
Luminal-binding protein 2	DDB_G0276445	3.12	1.69			3.08	3.39
Uncharacterized protein	DDB_G0272200	2.35	3.04	3.64	5.21		
p97	DDB_G0288065	2.07	3.16	2.31	1.01	6.10	2.80
**Hisactophilin-2**	**DDB_G0282143**	**1.23**	**1.48**			**2.00**	**1.35**
Eukaryotic peptide chain release factor	DDB_G0288613	1.13	1.45	2.36	2.83		
Putative glutamine synthetase type III	DDB_G0279591	0.56	1.90	3.63	4.38	3.96	4.89
Putative bifunctional amine oxidase	DDB_G0291301			4.91	3.28	2.88	1.41
cAMP-binding protein 1	DDB_G0272560			4.77	3.25	2.98	1.88
**Hisactophilin-1**	**DDB_G0282141**			**4.00**	**2.70**	**4.61**	**3.64**
Phosphoenolpyruvate carboxykinase	DDB_G0271678			3.23	3.45	3.68	5.07
V-type proton ATPase subunit G	DDB_G0277971			3.17	3.60	3.94	4.47
**Myosin-J heavy chain**	**DDB_G0272112**			**3.12**	**4.68**	**3.34**	**3.58**
ADP-ribosylation factor 1	DDB_G0289173			3.10	2.49	2.66	2.14
60S ribosomal protein L36	DDB_G0270984			3.05	4.64	2.97	3.30
Probable arginine–tRNA ligase	DDB_G0272867			3.02	4.17	1.33	1.46
Probable replication factor C subunit 3	DDB_G0293702			2.67	2.88	1.87	1.72
Ran binding protein 1 domain-containing protein	DDB_G0287391			2.52	2.98	2.88	2.86
Lysine–tRNA ligase	DDB_G0281437			2.47	2.93	1.86	1.36
Putative rho GDI 1	DDB_G0291077			2.30	2.23	1.74	1.35
Probable aconitate hydratase	DDB_G0278779			2.21	2.88	1.80	1.43
Probable pyridoxal 5-phosphate synthase subunit pdx1	DDB_G0288299			2.07	1.31	3.23	2.15
Vacuolar protein sorting-associated protein 29	DDB_G0288787			2.03	1.35	1.87	1.30
Probable serine/threonine-protein kinase	DDB_G0277449			2.01	1.93	1.61	1.50
**Actin-binding protein F**	**DDB_G0291229**			**1.90**	**2.61**	**1.64**	**1.64**
Small aggregate formation protein	DDB_G0287587			1.89	2.56	2.50	2.80
Vacuolin-A	DDB_G0289485			1.76	4.33	1.75	2.44
Cysteine proteinase 5	DDB_G0272815			1.74	1.77	2.34	2.49
3-oxoacid CoA-transferase	DDB_G0288105			1.73	4.06	1.54	3.28
**Dynamin-A**	**DDB_G0277849**			**1.69**	**3.03**	**1.87**	**3.14**
**LIM domain-containing protein E**	**DDB_G0279415**			**1.63**	**1.98**	**1.23**	**1.50**
Uncharacterized protein	DDB_G0276473			1.44	1.53	1.36	1.44
Uncharacterized protein	DDB_G0288133			1.38	3.88	0.76	1.96
Vacuolar proton translocating ATPase 100 kDa subunit	DDB_G0291858			1.30	1.55	1.58	1.93
**Drebrin-like protein**	**DDB_G0273447**			**1.20**	**3.24**	**1.15**	**2.87**
Bifunctional purine biosynthesis protein	DDB_G0277087			1.16	1.53	1.78	1.95

*Proteins are ordered based on detection in the different experiments and fold change (FC), from largest to smallest. Proteins associated with the cytoskeleton are in bold. DDB_G ID, gene ID (http://dictybase.org). FC, log2 fold change; P, −log10 p-value.*

## Discussion

The ubiquitin X regulatory domain (UBXD) containing protein family is present in all eukaryotes and is the largest subgroup of p97 cofactors (Rijal et al., 2016). In *A. thaliana* fifteen, in human thirteen and in *D. discoideum* eleven largely undescribed UBXD proteins have been identified (Buchberger et al., 2001; Rijal et al., 2016). The UBXD9 subfamily is particularly interesting as its members are so far the only identified p97 interacting proteins, which are able to regulate the oligomeric status of p97. Prominent members are ASPL (TUG) in *H. sapiens*, PUX1 in *A. thaliana*, and UBXD9 in *D. discoideum* (Rancour et al., 2004; Orme and Bogan, 2012; Rijal et al., 2016). UBXD9 orthologs are also present in flies, worms, fungi and other eukaryotes (Figure 2). The goal of the present study was the characterization of the functional regions in *D. discoideum* UBXD9 through analysis of full-length and truncated UBXD9 (Figure 3A) and the identification of novel UBXD9 interaction partners through IP experiments and proximity labeling proteomics.

The multiple sequence alignment showed significant homology between all UBXD9 members in the N-terminal UBL1 and LHU domains, in the middle coiled coil region and in the C-terminal UBX domain. All regions in between are not well conserved (Figure 2). *A. thaliana* UBXD9 was omitted from the alignment, because this protein has only about half the size of the other UBXD9 orthologs and lacks the UBL1 and LHU domains (Rancour et al., 2004). The UBL domain is commonly found in proteins that can interact with the proteasome (Hartmann-Petersen and Gordon, 2004). All UBXD9 proteins harbor the eponymous UBX domain in the C-terminal part, which comprises about 80 amino acids. For *A. thaliana* UBXD9 it was shown that a C-terminal construct harboring the UBX domain and the complete C-terminus (named UBX-C) was able to interact with p97, while the UBX domain alone was not (Rancour et al., 2004). For human UBXD9 two p97 binding regions have been proposed: a region which contains a central SHP box and another region which harbors the conserved central coiled coil domain, the UBX domain and the C-terminal region (Orme and Bogan, 2012). The SHP box is composed of eight amino acid with the sequence (FXGXGQRU, where X is any amino acid and U is a non-polar residue) and was originally identified in the yeast Derlin-1 homolog as p97 binding motif (Yeung et al., 2008). The SHP box was identified in human and mouse UBXD9 but is not detectable in *D. melanogaster*, *C. elegans*, *S. cerevisiae*, and *D. discoideum* UBXD9. The interpretation of the published interactions of human UBXD9 truncation constructs with p97 is complicated, as also an N-terminal construct harboring amino acids 1–237, which neither contained the SHP box nor the UBX domain, was found to bind to p97. On the other hand, a construct equivalent to *A. thaliana* UBX-C (see above) did not interact with p97 (Orme and Bogan, 2012). Recent structural studies suggest that the SHP box is dispensable for binding of human UBXD9 to p97 and confirm the importance of the UBX domain for the interaction of *H. sapiens* and *A. thaliana* UBXD9 with p97 (Arumughan et al., 2016; Banchenko et al., 2019). The UBX domain adopts the common β_1_-β_2_-α_1_-β_3_-β_4_-α_2_-β_5_ secondary structure, as originally described for ubiquitin (Buchberger et al., 2001). The structures of the ND1 domains of p97 in complex with truncated human and *A. thaliana* UBXD9 revealed, that the highly conserved β_3_/β_4_ loop of the UBX domain inserts into a gap between the p97 N_a_ and N_b_ subdomains. The loop forms a β-turn containing four residues with two proline residues in position two and three, which are also conserved in *D. discoideum* UBXD9 (Supplementary Figure 1). The loop adopts a rare *cis*-Pro touch-turn motif in human and *A. thaliana* UBXD9 which is critical for the interaction with the p97 N domain (Arumughan et al., 2016; Banchenko et al., 2019). We found, that all *D. discoideum* UBXD9 truncation constructs, that contained the UBX domain were able to bind to p97 and that, in contrast to results obtained with human and *A. thaliana* UBXD9, the UBX domain is necessary and sufficient for the interaction of *D. discoideum* UBXD9 with p97 (Figure 3B and Table 1; Rancour et al., 2004; Orme and Bogan, 2012). Site directed mutagenesis followed by interaction studies will show whether the two proline residues in the β_3_/β_4_ loop of *D. discoideum* UBXD9 are pivotal for the interaction of this protein with p97.

The 3D structures of human p97^ND1^ with human UBXD9^313–500^ and *A. thaliana* UBXD9^39–212^ were also very informative with respect to the hexamer disassembling activity of these proteins (Arumughan et al., 2016; Banchenko et al., 2019). The structures revealed a helical lariat N-terminal of the UBX domain, which embraces the N domain and consists of the α-helices α_–__1_ and α_0_, an unordered region, and the closing beta strand β_0_ (Figure 4C). It was found, that the extended *H. sapiens* UBX domain with the complete β_0_-α_–__1_-α_0_ lariat encompassing amino acids 313–500 was sufficient to trigger the disassembly of p97 hexamers (Arumughan et al., 2016). Notably, this construct did not contain the SHP box which was previously suggested to be important for interaction with p97 (Orme and Bogan, 2012). Our analyses showed that only full-length UBXD9 and UBXD9^261–573^ had p97 hexamer disassembly activity, while UBXD9^331–573^ and UBXD9^384–573^ could bind to but failed to disassemble p97 hexamers (Figures 3B,C, 4A,B). Structure based modeling of *D. discoideum* p97^ND1^ in complex with these three truncation constructs revealed that only UBXD9^261–573^ contains the complete helical lariat, while UBXD9^331–573^ lacks the closing β_0_ strand and UBXD9^384–573^ misses the complete β_0_-α_–__1_-α_0_ lariat (Figures 4D–F).

Since UBXD9 family proteins regulate the oligomeric status of p97, it is feasible that they interact with p97 as oligomers. This possibility is still largely unexplored. Based on velocity sedimentation centrifugation *A. thaliana* UBXD9 was reported to be likely monomeric (Rancour et al., 2004). This protein is a short version of UBXD9 family members, it lacks the complete N-terminal half and does not contain the conserved coiled coil domain of other UBXD9 family members (Figure 2; Rancour et al., 2004). We found, that full-length *D. discoideum* UBXD9 as well as UBXD9^261–573^, UBXD9^331–573^, and UBXD9^384–573^, the latter harboring only the second, C-terminal coiled coil domain, oligomerize into dimers or trimers and hexamers (Figure 5). Oligomerization is often mediated by coiled coil domains (Lupas and Bassler, 2017) and these proteins contain with exception of UBXD9^384–573^ the conserved coiled coil domain in the central part (aa 343–375) and a second coiled coil domain in the C-terminal region (aa 503–549) downstream of the UBX domain (Figure 3A). Since the conserved coiled coil domain in the central part is absent in UBXD9^384–573^, we propose that the C-terminal coiled coil domain is responsible for the oligomerization of *D. discoideum* UBXD9. Notably, this domain appears to be absent from the other UBXD9 family members and it is at present not clear whether these other members interact with p97 as monomers, whether they oligomerize via a different region or whether the C-terminal region mediates oligomerization despite the lack of a coiled coil element.

**FIGURE 8:**
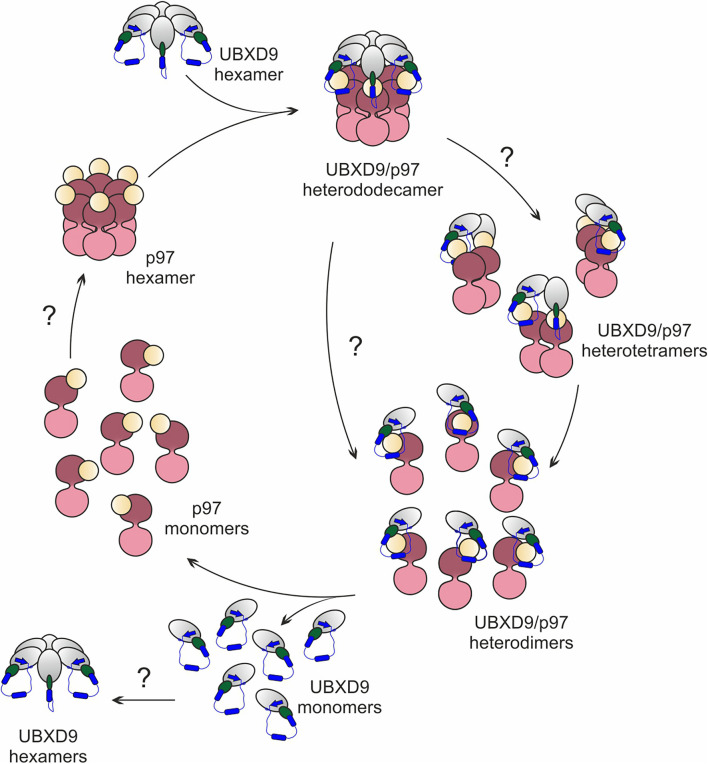
Structure-based model for the disassembly of the p97 hexamer by UBXD9. We propose, that a *D. discoideum* UBXD9 hexamer binds to a p97 hexamer and forms in the first step a hetero-dodecamer. The course of the dissociation of this complex is still unclear and possibly proceeds via intermediate steps. This could result in hetero-tetramers, which then dissociate to heterodimers and finally UBXD9 and p97 monomers are released. These are then free for another round of oligomerization. The p97 D1 and D2 domains are depicted in dark and light pink, respectively, and the N domain in yellow. UBXD9 is shown schematically with the UBX domain in green. The blue cylinders represent the α_0_ and α_–__1_ helices and the blue arrow the β_0_ strand.

Based on published structures of truncated human and *A. thaliana* UBXD9 in complex with p97^ND1^ (Arumughan et al., 2016; Banchenko et al., 2019), our structural modeling, and biochemical results, we propose a model for the interaction with and the disassembly of p97 by *D. discoideum* UBXD9 (Figure 8). The protein has the capacity to oligomerize (Figure 5) and likely interacts as a hexamer with the p97 hexamer, leading to the formation of a hetero-dodecamer. Binding is mediated by the UBX domain and the N-terminal extension of the UBX domain, a β_0_-α_–__1_-α_0_ lariat structure, embraces the N-domain of p97 and presumably destabilizes the D1:D1’ intermonomeric interface (Figures 4C–F; Arumughan et al., 2016). The course of the dissociation of this complex is still unclear; however, one can speculate that dissociation proceeds via intermediate steps, as it is apparently the case in *H. sapiens* and *A. thaliana*, where hetero-tetramers and hetero-dimers have been observed (Arumughan et al., 2016; Banchenko et al., 2019). Thus, dissociation could result in hetero-hexamers or hetero-tetramers, then hetero-dimers and finally UBXD9 and p97 monomers will be released. These are then free for another round of oligomerization (Figure 8). To date, nothing is known about the regulation of UBXD9 in these complex interactions with p97.

Little is also known so far about UBXD9 interacting proteins other than p97. For *A. thaliana* UBXD9 it was even speculated that p97 might be the only interaction partner (Rancour et al., 2004). We applied three different experimental approaches to identify UBXD9 interacting proteins, immune-precipitations with i) UBXD9-GFP and ii) GFP-UBXD9 expressing cells followed by mass spectrometry and iii) proximity labeling proteomics. Our results confirmed the self-association of UBXD9 and its interaction with p97, supporting the functionality, specificity, and efficacy of our approach. In total, we identified 185 potential UBXD9 binding proteins (Figure 6E and Supplementary Tables 1–3). We detected only three proteins, namely p97, UBXD9 and the putative glutamine synthetase type III (GSIII), in each of the three approaches (Table 2). In this context, it has recently been reported that p97 promotes the degradation of glutamine synthetase (GS), a homodecamer, in human cells (Nguyen et al., 2017). We currently do not know which functional consequence direct or indirect binding of *D. discoideum* UBXD9 to GSIII, a homododecamer, may have. An attractive possibility is that it may be involved in its turnover together with p97.

Of particular interest are also the 33 proteins, which we identified in two of our three experimental approaches (Figure 6E and Table 2). GO analysis revealed a strong enrichment of the actin cytoskeleton, the phagosome and the lysosome (Figure 7 and Table 2). Among these 33 proteins are myosin J heavy chain, a processive F-actin motor protein (Jung et al., 2009), dynamin A, involved in phagosome maturation (Gopaldass et al., 2012), and five additional actin cytoskeletal proteins; thus, UBXD9 appears to be involved in dynamic actin cytoskeletal processes—a notion that is further supported by the involvement of human UBXD9 in dynamic cellular processes, in Golgi reassembly and the redistribution of GLUT4 storage vesicles (Orme and Bogan, 2012; Habtemichael et al., 2018). A further UBXD9 interacting protein of interest was the ER chaperone Grp78 (luminal binding protein 2, BiP) (Table 2). Their interaction may be doubtful, as Grp78 localizes to the ER lumen and UBXD9 to the nucleus and cytoplasm (Rijal et al., 2016; Elfiky et al., 2020). However, in response to ER stress a fraction of Grp78 escapes the ER and translocates to the cytoplasm and the plasma membrane, providing an opportunity for the interaction with UBXD9 (Elfiky et al., 2020). Recently, it was shown that *D. discoideum* Grp78 and p97 were up-regulated in response to ER stress (Dominguez-Martin et al., 2018). Thus, it is conceivable that UBXD9 plays a role in the ER stress response together with p97, Grp78 and possible additional factors.

## Conclusion

p97, an abundant homohexameric AAA + ATPase, is highly conserved from *D. discoideum* to man and plays a central role in cellular protein homeostasis. Its regulation is subject to more than 30 cofactors. The largest family of cofactors is the Ubiquitin Regulatory X (UBX) domain protein family. Members of the UBXD9 subfamily are so far the only identified p97 interacting proteins, which are able to regulate the oligomeric status of p97. We could show that the UBX domain of *D. discoideum* UBXD9 is necessary and sufficient for the interaction with p97. An N-terminal extension of the UBX domain, which folds into a β_0_-α_–__1_-α_0_ lariat structure, is required for the dissociation of p97 hexamers. We propose that hexamers of UBXD9 interact with p97 hexamers and disrupt the p97 subunit interactions via destabilization of the p97 D1:D1’ intermolecular interface. Immune-precipitations with UBXD9-GFP and GFP-UBXD9 expressing cells as well as proximity labeling proteomics confirmed the self-association of UBXD9 and its interaction with p97. In addition, we identified through this approach 183 novel putative UBXD9 interacting proteins, among them are several cytoskeletal proteins, the glutamine synthetase type III (GSIII) and the ER luminal binding protein 2 (also known as BiP or Grp78).

## Data Availability Statement

The datasets presented in this study can be found in online repositories. The names of the repository/repositories and accession number(s) can be found below: https://www.ebi.ac.uk/pride/archive/projects/PXD027160; https://www.ebi.ac.uk/pride/archive/projects/PXD027162.

## Author Contributions

JR designed and performed experiments, analyzed the data, and drafted the manuscript and figures. RR and LN performed experiments and contributed to reagents. AH performed secondary structure predictions and the molecular modeling. LE and CC designed the experiments. LE analyzed and reviewed all data, and prepared the final version of the manuscript. All authors approved the final version of the manuscript.

## Conflict of Interest

The authors declare that the research was conducted in the absence of any commercial or financial relationships that could be construed as a potential conflict of interest.

## Publisher’s Note

All claims expressed in this article are solely those of the authors and do not necessarily represent those of their affiliated organizations, or those of the publisher, the editors and the reviewers. Any product that may be evaluated in this article, or claim that may be made by its manufacturer, is not guaranteed or endorsed by the publisher.

## Footnotes

1
http://proteomics.cecad-labs.uni-koeln.de/Protocols.955.0.html

2
http://www.proteomexchange.org/

## Supplementary Material

Supplementary Figure 1 Structure-based amino acid sequence alignment of human ASPL (UBXD9) and *D. discoideum* UBXD9. Secondary structure elements are color mapped; (α-helices: green, β-strands: red; cysteine residues: yellow). The UBL domain, the UBX domain, the helical lariat and the coiled coil (CC) regions are indicated by rectangles above and below the sequence alignment. Potential coiled coil motifs were predicted using MultiCoil (Wolf et al., 1997). The conserved aspartate (D) of the helical lariat and the double proline (PP) motif are boxed. Amino acid positions indicated in blue above and below the amino acid sequences denote the limits of various experimental constructs. Genbank accession numbers are XP_641771 for *D. discoideum* and NP_076988 for human UBXD9.

Supplementary Figure 2 Pull-down experiments with recombinant p97 and full-length or truncated GST-UBXD9 coupled to glutathione beads. GST coupled to glutathione beads was used as negative control. Representative SDS PAGE gels stained with Coomassie Blue. The position of p97 is indicated. *Positions of GST and of full-length UBXD9 and UBXD9 truncation constructs tagged with GST.

Supplementary Figure 3 UBXD9 and UBXD9^261–573^ reduce the ATPase activity of p97. Data is presented as relative ATPase activity, mean values and SD of three experiments. For statistical analysis, the Dunnett’s multiple comparison test, implemented in GraphPad Prism as *post hoc* analysis, was performed. ****p* ≤ 0.001.

Supplementary Figure 4 Analysis of IP experiments and proximity labeling proteomics. **(A)** GFP trap experiments with the soluble proteins from total cell lysates of AX2 cells expressing GFP, UBXD9-GFP, or GFP-UBXD9. Top: SDS-PAGE and silver stain of proteins bound to the beads (Pellet). *Indicates the position of GFP-UBXD9 and # of GFP. +Indicates the position of the absent UBXD9-GFP silver band and of the visible UBXD9-GFP immunoblot band (see Western Blot). Bottom: Western blotting of proteins bound to the beads (Pellet). Endogenous UBXD9 of 95 kDa and GFP-tagged UBXD9 of 120 kDa were detected with the polyclonal UBX23520 antibody. *Indicates the position of GFP-tagged UBXD9 and o of untagged UBXD9. **(B)** BioID experiments with soluble proteins from total cell lysates of AX2 and AX2/BirA-UBXD9 cells. Top: SDS-PAGE and silver stain of soluble proteins before (S^1^) and after (S^2^) incubation with streptavidin sepharose beads and proteins bound to the beads (Pellet). The position of BirA-UBXD9 is indicated. Bottom: Western blotting of soluble proteins after (S^2^) incubation with streptavidin sepharose beads and of proteins bound to the beads (Pellet). Endogenous UBXD9 and BirA-tagged UBXD9 were detected with the polyclonal UBX23520 antibody (top panel) and p97 with the polyclonal p97_8_6842 antibody (lower panel).
