# Supplementary figures and images for: Domain Organization of the UBX Domain Containing Protein 9 and Analysis of Its Interactions With the Homohexameric AAA + ATPase p97 (Valosin-Containing Protein)
Source: Front Cell Dev Biol. 2021 Sep 23;9:748860. doi: 10.3389/fcell.2021.748860 (PMC8495200; doi:10.3389/fcell.2021.748860)

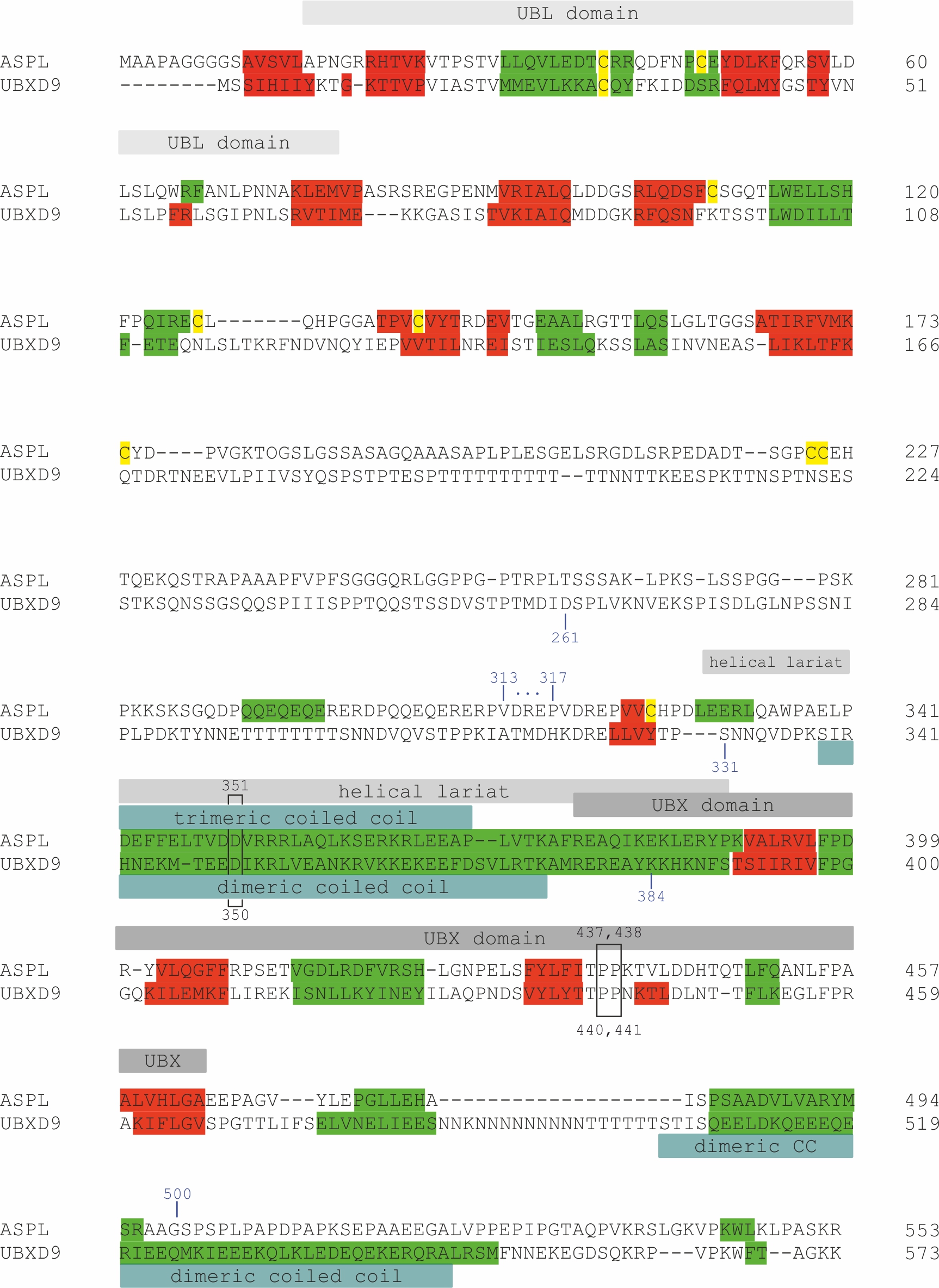

Supplement: Supplementary Figure 1 — Structure-based amino acid sequence alignment of human ASPL (UBXD9) and D. discoideum UBXD9. Secondary structure elements are color mapped; (α-helices: green, β-strands: red; cysteine residues: yellow). The UBL domain, the UBX domain, the helical lariat and the coiled coil (CC) regions are indicated by rectangles above and below the sequence alignment. Potential coiled coil motifs were predicted using MultiCoil (Wolf et al., 1997). The conserved aspartate (D) of the helical lariat and the double proline (PP) motif are boxed. Amino acid positions indicated in blue above and below the amino acid sequences denote the limits of various experimental constructs. Genbank accession numbers are XP_641771 for D. discoideum and NP_076988 for human UBXD9. [file Image_1.jpg]

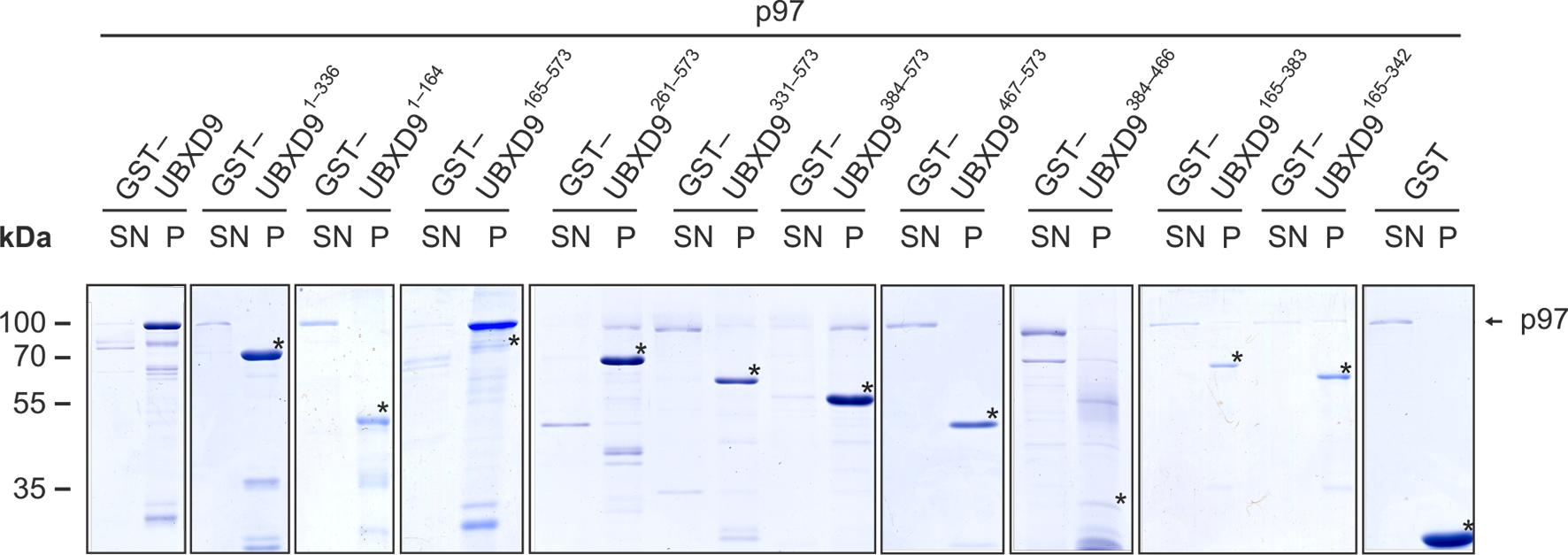

Supplement: Supplementary Figure 2 — Pull-down experiments with recombinant p97 and full-length or truncated GST-UBXD9 coupled to glutathione beads. GST coupled to glutathione beads was used as negative control. Representative SDS PAGE gels stained with Coomassie Blue. The position of p97 is indicated. *Positions of GST and of full-length UBXD9 and UBXD9 truncation constructs tagged with GST. [file Image_2.TIF]

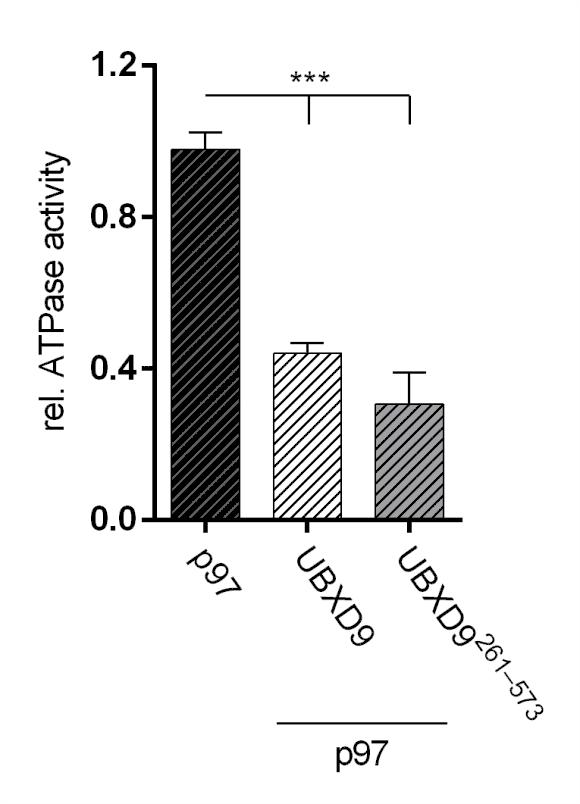

Supplement: Supplementary Figure 3 — UBXD9 and UBXD9261–573 reduce the ATPase activity of p97. Data is presented as relative ATPase activity, mean values and SD of three experiments. For statistical analysis, the Dunnett’s multiple comparison test, implemented in GraphPad Prism as post hoc analysis, was performed. ***p ≤ 0.001. [file Image_3.TIF]

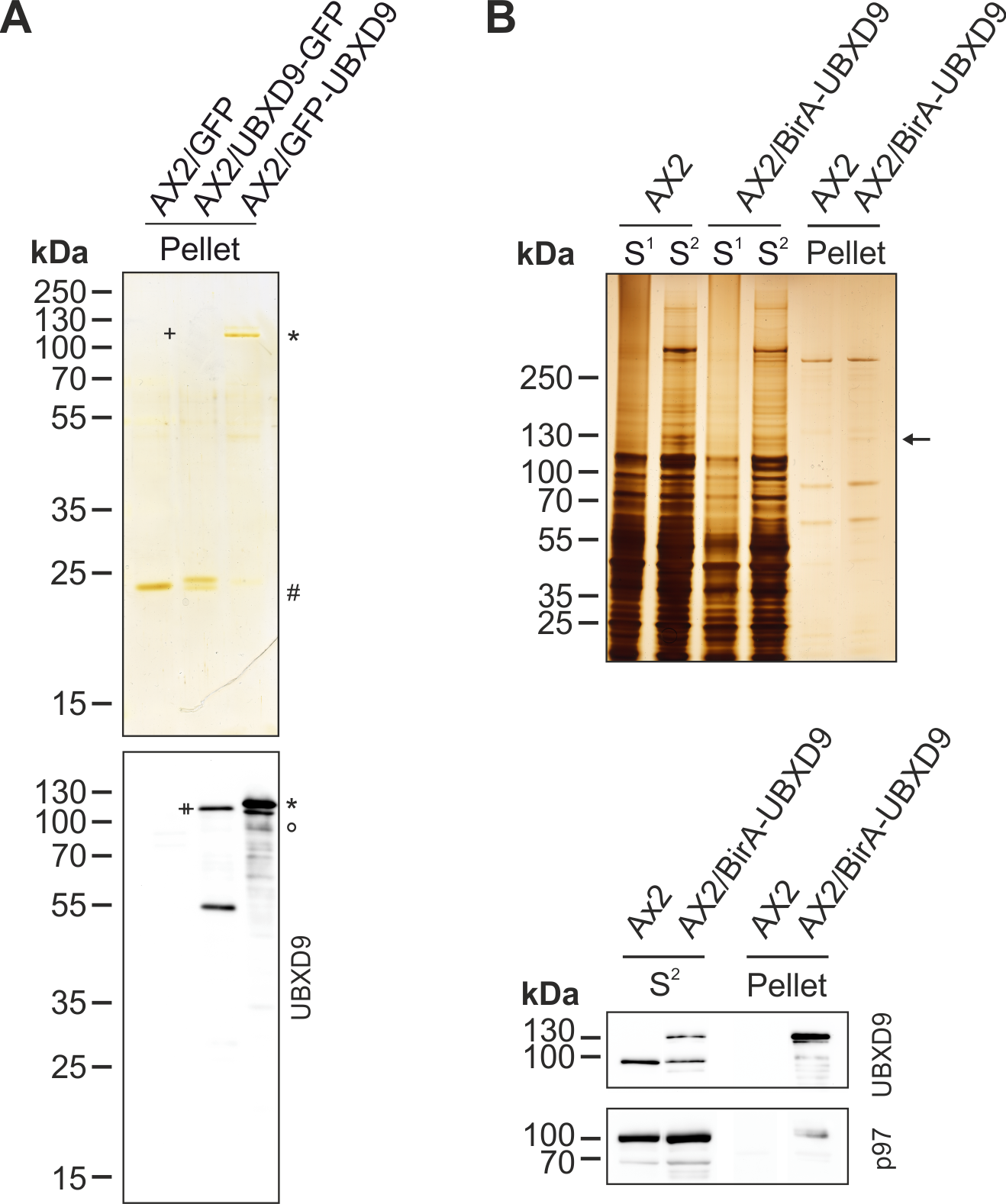

Supplement: Supplementary Figure 4 — Analysis of IP experiments and proximity labeling proteomics. (A) GFP trap experiments with the soluble proteins from total cell lysates of AX2 cells expressing GFP, UBXD9-GFP, or GFP-UBXD9. Top: SDS-PAGE and silver stain of proteins bound to the beads (Pellet). *Indicates the position of GFP-UBXD9 and # of GFP. +Indicates the position of the absent UBXD9-GFP silver band and of the visible UBXD9-GFP immunoblot band (see Western Blot). Bottom: Western blotting of proteins bound to the beads (Pellet). Endogenous UBXD9 of 95 kDa and GFP-tagged UBXD9 of 120 kDa were detected with the polyclonal UBX23520 antibody. *Indicates the position of GFP-tagged UBXD9 and o of untagged UBXD9. (B) BioID experiments with soluble proteins from total cell lysates of AX2 and AX2/BirA-UBXD9 cells. Top: SDS-PAGE and silver stain of soluble proteins before (S1) and after (S2) incubation with streptavidin sepharose beads and proteins bound to the beads (Pellet). The position of BirA-UBXD9 is indicated. Bottom: Western blotting of soluble proteins after (S2) incubation with streptavidin sepharose beads and of proteins bound to the beads (Pellet). Endogenous UBXD9 and BirA-tagged UBXD9 were detected with the polyclonal UBX23520 antibody (top panel) and p97 with the polyclonal p97_8_6842 antibody (lower panel). [file Image_4.TIF]
