# Supplementary material for: Domain Organization of the UBX Domain Containing Protein 9 and Analysis of Its Interactions With the Homohexameric AAA + ATPase p97 (Valosin-Containing Protein)
Source: Front Cell Dev Biol. 2021 Sep 23;9:748860. doi: 10.3389/fcell.2021.748860 (PMC8495200; doi:10.3389/fcell.2021.748860)
Supplement: Supplementary file 5 [file Data_Sheet_1.PDF]

Table S1: Significantly co-purified proteins in AX2/ UBXD9-GFP versus AX2/GFP.
FC ≥ 1.4, p-value ≤ 0.05.

Protein Description	DDB_G ID	Log2 FC	-Log10 P-value
CBS domain-containing protein	DDB_G0289609	3.39	5.41
Uncharacterised protein	DDB_G0272200	3.64	5.21
Myosin-J heavy chain	DDB_G0272112	3.12	4.68
60S ribosomal protein L36a	DDB_G0270984	3.05	4.64
Probable methylmalonate-semialdehyde dehydrogenase, mitochondrial	DDB_G0289085	2.19	4.41
Type-3 glutamine synthetase	DDB_G0279591	3.63	4.38
Peter Pan-like protein	DDB_G0285515	2.21	4.37
Vacuolin-A	DDB_G0289485	1.76	4.33
Rac guanine nucleotide exchange factor JJ	DDB_G0275679	2.37	4.21
Putative rRNA methyltransferase	DDB_G0284945	4.92	4.18
Probable arginine--tRNA ligase, cytoplasmic	DDB_G0272867	3.02	4.17
Probable succinyl-CoA:3-ketoacid coenzyme A transferase, mitochondrial	DDB_G0288105	1.73	4.06
Uncharacterised protein	DDB_G0288133	1.38	3.88
Uncharacterised protein	DDB_G0290085	3.21	3.86
Clustered mitochondria protein homolog	DDB_G0292806	3.26	3.78
Putative uncharacterised protein	DDB_G0271804	3.33	3.77
Actin-related protein 2/3 complex subunit 3	DDB_G0292804	2.18	3.66
V-type proton ATPase subunit G	DDB_G0277971	3.17	3.60
Uncharacterissed protein	DDB_G0278025	3.50	3.60
Protein transport protein SEC31	DDB_G0270992	2.27	3.47
Phosphoenolpyruvate carboxykinase (ATP)	DDB_G0271678	3.23	3.45
Ras-related protein RabC	DDB_G0271736	2.96	3.42
Kinesin-related protein 1	DDB_G0290963	3.24	3.40
Uncharacterised protein	DDB_G0287709	4.51	3.38
Histone H4	DDB_G0277183	1.66	3.36
Putative bifunctional amine oxidase	DDB_G0291301	4.91	3.28
cAMP-binding protein 1	DDB_G0272560	4.77	3.25
Drebrin-like protein	DDB_G0273447	1.20	3.24
Uncharacterised protein	DDB_G0272989	1.04	3.21
Uncharacterised protein	DDB_G0271816	2.54	3.19
Uncharacterised protein	DDB_G0290181	2.52	3.10
Dynamin-A	DDB_G0277849	1.69	3.03
Clathrin heavy chain	DDB_G0277221	1.19	3.01
Ran-specific GTPase-activating protein homolog	DDB_G0287391	2.52	2.98
Lysine--tRNA ligase	DDB_G0281437	2.47	2.93
Probable aconitate hydratase, mitochondrial	DDB_G0278779	2.21	2.88
Probable replication factor C subunit 3	DDB_G0293702	2.67	2.88
Ras-like protein rasB	DDB_G0292998	3.00	2.87
Eukaryotic peptide chain release factor subunit 1	DDB_G0288613	2.36	2.83
Hisactophilin-1	DDB_G0282141	4.00	2.70
RuvB-like helicase	DDB_G0293226	2.16	2,68
Uncharacterised protein	DDB_G0290465	1.74	2.67

Adenylyl cyclase-associated protein	DDB_G0288769	1.22	2.67
Formin-H	DDB_G0285589	2.76	2.66
32 kDa heat shock protein	DDB_G0272819	1.71	2.64
Actin-binding protein F	DDB_G0291229	1.90	2.61
Uncharacterised protein	DDB_G0270026	1.93	2.58
Small aggregate formation protein	DDB_G0287587	1.89	2.56
Ras-related protein Rab-4	DDB_G0292406	1.83	2.54
Uncharacterised protein	DDB_G0269334	4.60	2.52
Uncharacterised protein	DDB_G0288041	2.25	2.51
Methenyl tetrahydrofolate cyclohydrolase	DDB_G0280291	1.11	2.50
ADP-ribosylation factor 1	DDB_G0289173	3.10	2.49
Probable inactive serine/threonine-protein kinase	DDB_G0280131	1.41	2.46
Uncharacterised protein	DDB_G0293070	2.31	2.46
Acetyl-CoA carboxylase	DDB_G0288387	0.52	2.43
Uncharacterised protein	DDB_G0277009	2.66	2.40
Uncharacterised protein	DDB_G0281925	2.76	2.34
Uncharacterised protein	DDB_G0348940	2.55	2.31
Uncharacterised protein	DDB_G0274655	2.18	2.30
Uncharacterised protein	DDB_G0290597	2.31	2.29
Bifunctional purine synthesis protein purC/E	DDB_G0283987	1.28	2.29
Unclassified GTPase	DDB_G0294292	2.06	2.28
Serine/threonine-protein kinase rio1	DDB_G0280431	2.91	2.26
Putative glutathione S-transferase alpha-2	DDB_G0272632	3.74	2.26
Uncharacterised protein	DDB_G0280801	2.29	2.24
Ras-related protein Rab-1D	DDB_G0284985	2.05	2.24
Putative rho GDP-dissociation inhibitor 1	DDB_G0291077	2.30	2.23
Ribosomal protein L1 family protein	DDB_G0267850	1.86	2.22
Protein PYR1-3	DDB_G0276335	0.60	2.21
Ribosome biogenesis regulatory protein homolog	DDB_G0274521	2.32	2.17
Dynamin-like protein B	DDB_G0285931	2.15	2.15
60S ribosomal protein L37	DDB_G0285971	0.95	2.12
Eukaryotic translation initiation factor 6	DDB_G0276493	1.04	2.02
LIM domain-containing protein E	DDB_G0279415	1.63	1.98
Uncharacterised protein	DDB_G0276105	2.41	1.98
Uncharacterised protein	DDB_G0284845	1.40	1.95
Probable serine/threonine-protein kinase	DDB_G0277449	2.01	1.93
40S ribosomal protein S4	DDB_G0272825	1.29	1.93
Maestro heat-like repeat-containing protein family member 1	DDB_G0291161	0.88	1.91
Ras GTPase-activating-like protein gapA	DDB_G0269140	1.83	1.86
60S ribosomal protein L10	DDB_G0288273	0.55	1.79
Probable ATP-dependent RNA helicase ddx5	DDB_G0293036	1.40	1.79
Cysteine proteinase 5	DDB_G0272815	1.74	1.77
Dynamin-B	DDB_G0277851	1.47	1.76
Probable ATP-citrate synthase	DDB_G0278345	0.86	1.71
Probable GH family 25 lysozyme 2	DDB_G0274181	1.38	1.71
Uncharacterised protein	DDB_G0278685	1.82	1.66
Actin-related protein 2/3 complex subunit 1	DDB_G0277825	1.09	1.65

Small glutamine-rich tetratricopeptide repeat-containing protein	DDB_G0280345	1.83	1.63
60S ribosomal protein L26	DDB_G0283741	0.43	1.61
PH and Rap-GAP domain-containing protein	DDB_G0271806	1.80	1.59
KRR1 small subunit processome component homolog	DDB_G0280805	1.90	1.59
NHP2-like protein 1 homolog	DDB_G0282243	2.69	1.58
Uncharacterised protein	DDB_G0290857	1.14	1.58
Uncharacterised protein	DDB_G0289833	0.96	1.57
Vegetative-specific protein V4	DDB_G0280533	2.56	1.57
Calcium-binding EF-hand domain-containing protein	DDB_G0289563	1.21	1.55
Vacuolar proton translocating ATPase 100 kDa subunit	DDB_G0291858	1.30	1.55
Uncharacterised protein	DDB_G0276473	1.44	1.53
Bifunctional purine biosynthesis protein purH	DDB_G0277087	1.16	1.53
Uncharacterised protein	DDB_G0272374	2.34	1.52
Uncharacterised protein	DDB_G0279285	5.67	1.49
Polyadenylate-binding protein 1-B	DDB_G0290745	1.06	1.48
Uncharacterised protein	DDB_G0275365	1.57	1.47
Uncharacterised protein	DDB_G0277957	0.75	1.47
ABC transporter F family member 4	DDB_G0267436	1.01	1.46
Uncharacterised protein	DDB_G0282303	1.13	1.41
RNA-binding protein pno1	DDB_G0287557	1.01	1.38
COP9 signalosome complex subunit 3	DDB_G0291848	1.35	1.38
Uncharacterised protein	DDB_G0290333	0.71	1.37
Talin-B	DDB_G0287505	1.48	1.37
Probable ATP-dependent RNA helicase ddx27	DDB_G0281711	2.41	1.35
Vacuolar protein sorting-associated protein 29	DDB_G0288787	2.03	1.35
Probable ATP-dependent RNA helicase ddx52	DDB_G0274325	1.39	1.34
Probable serine/threonine-protein kinase pdkA	DDB_G0281471	0.96	1.33
Protein SEC13 homolog	DDB_G0292052	0.51	1.32
Probable pyridoxal 5-phosphate synthase subunit pdx1	DDB_G0288299	2.07	1.31
Serine/threonine-protein kinase fray1	DDB_G0278863	1.18	1.30
p97	DDB_G0288065	2.31	1.01

Table S2: Significantly co-purified proteins in AX2/GFP-UBXD9 versus AX2/GFP.
FC ≥ 1.4, p-value ≤ 0.05.

Protein Description	DDB_G ID	Log2 FC	–Log10 P-value
Ras-related protein RabK3	DDB_G0290831	6.47	5.72
Phosphoenolpyruvate carboxykinase (ATP)	DDB_G0271678	3.68	5.07
Type-3 glutamine synthetase	DDB_G0279591	3.96	4.89
V-type proton ATPase subunit G	DDB_G0277971	3.94	4.47
Protein arginine N-methyltransferase 1	DDB_G0291556	3.28	4.20
Dual specificity protein kinase shkB	DDB_G0288617	3.49	3.90
Asparagine--tRNA ligase, cytoplasmic	DDB_G0275263	2.10	3.83
Ragulator complex protein LAMTOR5 homolog	DDB_G0283567	2.14	3.78
Hisactophilin-1	DDB_G0282141	4.61	3.64
Myosin-J heavy chain	DDB_G0272112	3.34	3.58
Luminal-binding protein 2	DDB_G0276445	3.08	3.39
60S ribosomal protein L36a	DDB_G0270984	2.97	3.30
Probable succinyl-CoA:3-ketoacid coenzyme A transferase, mitochondrial	DDB_G0288105	1.54	3.28
Dynamin-A	DDB_G0277849	1.87	3.14
26S proteasome non-ATPase regulatory subunit 6	DDB_G0270188	2.03	3.05
Uncharacterised protein	DDB_G0290603	3.12	2.99
Heat shock cognate 70 kDa protein 1	DDB_G0269144	1.57	2.90
Drebrin-like protein	DDB_G0273447	1.15	2.87
Ran-specific GTPase-activating protein homolog	DDB_G0287391	2.88	2.86
Small aggregate formation protein	DDB_G0287587	2.50	2.80
Cell division cycle protein 48	DDB_G0288065	6.10	2.80
Heat shock cognate 70 kDa protein 2	DDB_G0273249	1.97	2.71
26S proteasome non-ATPase regulatory subunit 7	DDB_G0279633	1.74	2.68
Cysteine proteinase 5	DDB_G0272815	2.34	2.49
UTP--glucose-1-phosphate uridylyltransferase 2	DDB_G0277879	3.68	2.45
Vacuolin-A	DDB_G0289485	1.75	2.44
Uncharacterised protein	DDB_G0281823	1.53	2.42
Actin-related protein 2/3 complex subunit 5	DDB_G0288319	1.46	2.35
Probable pyridoxal 5-phosphate synthase subunit pdx2	DDB_G0288305	0.86	2.25
Glutamate dehydrogenase 2	DDB_G0280319	3.10	2.25
Rab GDP dissociation inhibitor	DDB_G0268034	2.71	2.24
Sulfate adenylyltransferase	DDB_G0291029	3.59	2.18
Probable pyridoxal 5-phosphate synthase subunit pdx1	DDB_G0288299	3.23	2.15
ADP-ribosylation factor 1	DDB_G0289173	2.66	2.14
Adenosylhomocysteinase	DDB_G0267418	1.62	1.96
Uncharacterised protein	DDB_G0288133	0.76	1.96
Heat shock 70 kDa protein, mitochondrial	DDB_G0293298	0.86	1.95
Bifunctional purine biosynthesis protein purH	DDB_G0277087	1.78	1.95
Uncharacterised protein	DDB_G0279285	7.34	1.94
Vacuolar proton translocating ATPase 100 kDa subunit	DDB_G0291858	1.58	1.93
cAMP-binding protein 1	DDB_G0272560	2.98	1.88

Cathepsin B	DDB_G0283921	1.86	1.74
Probable replication factor C subunit 3	DDB_G0293702	1.87	1.72
Probable histone-binding protein rbbD	DDB_G0282529	1.93	1.70
DNA primase large subunit	DDB_G0270442	0.85	1.69
ATP-dependent 6-phosphofructokinase	DDB_G0274111	1.52	1.66
Actin-binding protein F	DDB_G0291229	1.64	1.64
Acetyl-coenzyme A synthetase	DDB_G0277815	2.34	1.57
Protein disulfide-isomerase 1	DDB_G0276141	2.31	1.54
LIM domain-containing protein E	DDB_G0279415	1.23	1.50
Probable serine/threonine-protein kinase	DDB_G0277449	1.61	1.50
Cystatin-A1	DDB_G0291834	1.94	1.49
Probable arginine--tRNA ligase, cytoplasmic	DDB_G0272867	1.33	1.46
ATP synthase subunit epsilon, mitochondrial	DDB_G0276241	1.57	1.46
Autophagy-related protein 8	DDB_G0286191	1.59	1.46
Uncharacterised protein	DDB_G0276473	1.36	1.44
1-Cys peroxiredoxin	DDB_G0282517	1.62	1.44
Probable malate dehydrogenase 2, mitochondrial	DDB_G0292600	1.33	1.44
V-type proton ATPase subunit E	DDB_G0275701	0.56	1.43
Probable aconitate hydratase, mitochondrial	DDB_G0278779	1.80	1.43
Putative bifunctional amine oxidase	DDB_G0291301	2.88	1.41
Coronin-A	DDB_G0267382	1.08	1.37
Lysine--tRNA ligase	DDB_G0281437	1.86	1.36
Putative rho GDP-dissociation inhibitor 1	DDB_G0291077	1.74	1.35
Hisactophilin-2	DDB_G0282143	2.00	1.35
Actin-related protein 2	DDB_G0272106	0.49	1.35
Uncharacterised protein	DDB_G0291464	2.61	1.33
Uncharacterised protein	DDB_G0284997	1.36	1.33
Vacuolar protein sorting-associated protein 29	DDB_G0288787	1.87	1.30

Table S3: Significantly co-purified proteins in AX2/BirA-UBXD9 versus AX2.
FC ≥ 1.4, p-value ≤ 0.05.

Protein Description	DDB_G ID	Log2 FC	–Log10 P-value
AP-2 complex subunit alpha-2	DDB_G0273439	1.90	6.31
UBXD9	DDB_G0279285	9.23	6.75
Glycyl-tRNA synthetase	DDB_G0284583	2.73	3.55
Methylenetetrahydrofolate dehydrogenase	DDB_G0277725	1.96	3.46
Extracellular signal-regulated kinase 1	DDB_G0286353	3.33	3.42
Dynacortin	DDB_G0283767	2.70	3.36
Aspartate-tRNA ligase	DDB_G0272236	2.12	3.19
p97	DDB_G0288065	2.07	3.16
Uncharacterised protein	DDB_G0272200	2.35	3.04
Protein elaA	DDB_G0285831	2.01	2.96
Uncharacterised protein	DDB_G0268114	1.99	2.39
Mo25-like family protein	DDB_G0284307	4.04	2.36
Centractin	DDB_G0288937	2.50	2.33
Coatomer subunit alpha	DDB_G0267982	1.59	2.26
Amidophosphoribosyltransferase	DDB_G0274321	1.88	2.15
saccharopine dehydrogenase	DDB_G0285267	0.86	2.10
Rho GTPase-activating protein gacC	DDB_G0284571	2.57	2.10
Porin	DDB_G0271848	2.52	1.97
Type-3 glutamine synthetase	DDB_G0279591	0.56	1.90
putative delta-24-sterol methyltransferase	DDB_G0288907	4.87	1.85
V-type proton ATPase subunit d	DDB_G0273071	5.16	1.78
Luminal-binding protein 2	DDB_G0276445	3.12	1.69
Putative D-lactate dehydrogenase	DDB_G0281101	1.66	1.58
Myosin-ID light chain	DDB_G0277917	1.78	1.54
Fatty acyl-CoA synthetase A	DDB_G0269242	2.38	1.53
Guanylate kinase	DDB_G0279001	1.69	1.51
Hisactophilin-2	DDB_G0282143	1.23	1.48
Mitochondrial substrate carrier family protein N	DDB_G0293646	2.13	1.48
26S proteasome non-ATPase regulatory subunit 14	DDB_G0272566	1.55	1.47
Eukaryotic peptide chain release factor	DDB_G0288613	1.13	1.45
Mitochondrial-processing peptidase	DDB_G0288777	0.65	1.38
Uncharacterised protein	DDB_G0278545	2.80	1.37
Cytochrome c oxidase subunit 4	DDB_G0281393	1.96	1.36
Secreted protein A	DDB_G0278725	2.33	1.35
Rho-related protein rac1A	DDB_G0288613	1.90	1.34
